# Structural enzymology studies with the substrate 3*S*‐hydroxybutanoyl‐CoA: bifunctional MFE1 is a less efficient dehydrogenase than monofunctional HAD

**DOI:** 10.1002/2211-5463.13786

**Published:** 2024-03-08

**Authors:** Shruthi Sridhar, Tiila‐Riikka Kiema, Werner Schmitz, Mikael Widersten, Rik K. Wierenga

**Affiliations:** ^1^ Faculty of Biochemistry and Molecular Medicine University of Oulu Finland; ^2^ Biocenter Oulu University of Oulu Finland; ^3^ Theodor Boveri Institute of Biosciences (Biocenter) University of Würzburg Germany; ^4^ Department of Chemistry – BMC Uppsala University Sweden

**Keywords:** crotonase, crystal structure, dehydrogenase, dynamical properties, stopped‐flow

## Abstract

Multifunctional enzyme, type‐1 (MFE1) catalyzes the second and third step of the β‐oxidation cycle, being, respectively, the 2*E*‐enoyl‐CoA hydratase (ECH) reaction (N‐terminal part, crotonase fold) and the NAD^+^‐dependent, 3*S*‐hydroxyacyl‐CoA dehydrogenase (HAD) reaction (C‐terminal part, HAD fold). Structural enzymological properties of rat MFE1 (RnMFE1) as well as of two of its variants, namely the E123A variant (a glutamate of the ECH active site is mutated into alanine) and the BCDE variant (without domain A of the ECH part), were studied, using as substrate 3*S*‐hydroxybutanoyl‐CoA. Protein crystallographic binding studies show the hydrogen bond interactions of 3*S*‐hydroxybutanoyl‐CoA as well as of its 3‐keto, oxidized form, acetoacetyl‐CoA, with the catalytic glutamates in the ECH active site. Pre‐steady state binding experiments with NAD^+^ and NADH show that the *k*_on_ and *k*_off_ rate constants of the HAD active site of monomeric RnMFE1 and the homologous human, dimeric 3*S*‐hydroxyacyl‐CoA dehydrogenase (HsHAD) for NAD^+^ and NADH are very similar, being the same as those observed for the E123A and BCDE variants. However, steady state and pre‐steady state kinetic data concerning the HAD‐catalyzed dehydrogenation reaction of the substrate 3*S*‐hydroxybutanoyl‐CoA show that, respectively, the *k*_cat_ and *k*_chem_ rate constants for conversion into acetoacetyl‐CoA by RnMFE1 (and its two variants) are about 10 fold lower as when catalyzed by HsHAD. The dynamical properties of dehydrogenases are known to be important for their catalytic efficiency, and it is discussed that the greater complexity of the RnMFE1 fold correlates with the observation that RnMFE1 is a slower dehydrogenase than HsHAD.

## Graphical abstract

Enzyme kinetic studies of rat multifunctional enzyme, type‐1 (RnMFE1) (monomeric, two active sites: ECH, HAD) and human 3*S*‐hydroxyacyl‐CoA dehydrogenase (HsHAD) (dimeric, one active site: HAD) show that RnMFE1 is a slower dehydrogenase than HsHAD. It is discussed that the different assembly and predicted different flexibility properties of MFE1 (curved arrows) and HAD are related to their different kinetic properties.

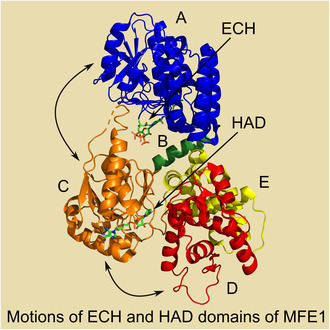

**Abbreviations**
AcAc‐CoA: acetoacetyl‐CoA also known as 3‐ketobutanoyl‐CoA being the oxidized form of 3*S*‐hydroxybutanoyl‐CoA
BCDE: the truncated variant of RnMFE1 starting at Ala260 (not having domain A)
E123A: the E123A point mutation variant of RnMFE1
ECH: 2*E*‐enoyl‐CoA hydratase
EDTA: ethylenediaminetetraacetic acid
HAD: 3*S*‐hydroxyacyl‐CoA dehydrogenase
HEPES: 4‐(2‐hydroxyethyl)‐1‐piperazineethanesulfonic acid
hlADH: horse liver alcohol dehydrogenase
HsHAD: human mitochondrial HAD
IPTG: isopropyl‐β‐1‐thio‐d‐galactopyranoside
MES: 2‐(N‐morpholino)ethanesulfonic acid
MFE1: multifunctional enzyme, type‐1
PIPES: piperazine‐N,N′‐bis(2‐ethanesulfonic) acid
RnMFE1: rat peroxisomal MFE1

The multifunctional enzyme, type‐1 (MFE1) is a monomeric, peroxisomal enzyme which catalyzes the 2*E*‐enoyl‐CoA hydratase reaction (ECH, EC4.2.1.17) and the subsequent NAD‐dependent 3*S*‐hydroxyacyl‐CoA dehydrogenase reaction (HAD, EC1.1.1.35) (Fig. 1) of the β‐oxidation cycle [1, 2, 3, 4]. MFE1 is present in plant and vertebrate peroxisomes, but not in yeast peroxisomes. The physiological substrate is not yet precisely known, but it has been shown to convert short‐, medium‐ and long‐chain unbranched 2*E*‐enoyl‐CoA substrate into 3‐ketoacyl‐CoA product [3]. Extensive structural characterization of rat peroxisomal MFE1 (RnMFE1, 72 kDa) has identified the five domains of MFE1 [5, 6], such that the ECH active site is formed by domains A and B and the HAD active site is formed by domains C, D and E (Fig. 2).

**Fig. 1:**
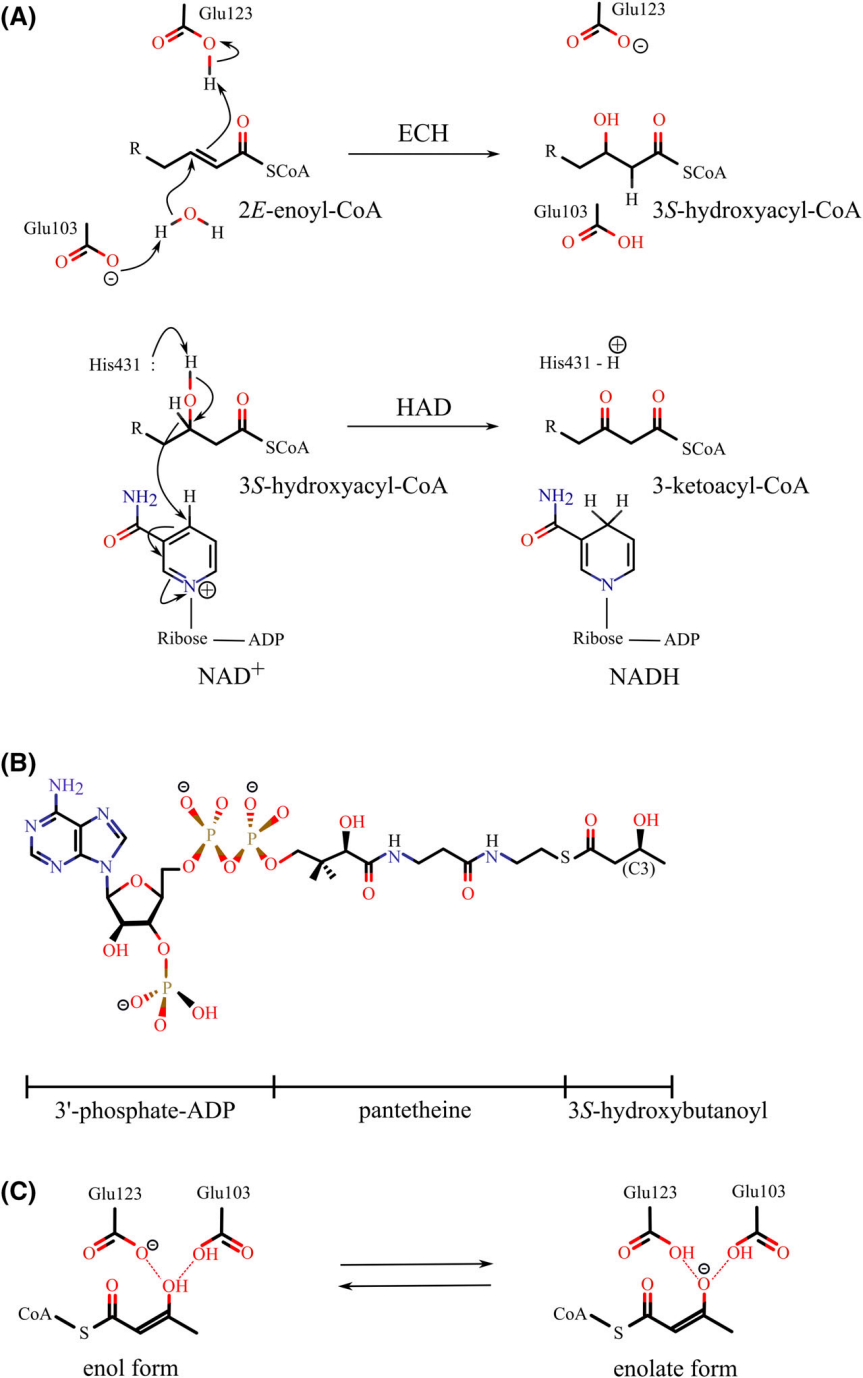
MFE1 catalyzes two subsequent reactions of the β‐oxidation cycle. (A) The reaction catalyzed by, respectively, the ECH and HAD active site of MFE1 ([13], reproduced with permission of the International Union of Crystallography). (B) The covalent structure of 3*S*‐hydroxybutanoyl‐CoA. (C) The 3‐keto group of AcAc‐CoA can be present in different states, due to keto‐enol tautomerization. Visualized in the left diagram is the protonation state of the catalytic glutamates of the ECH active site in the presence of the bound enol form of AcAc‐CoA, whereas the right diagram shows this protonation state in the presence of its bound enolate form.

**Fig. 2:**
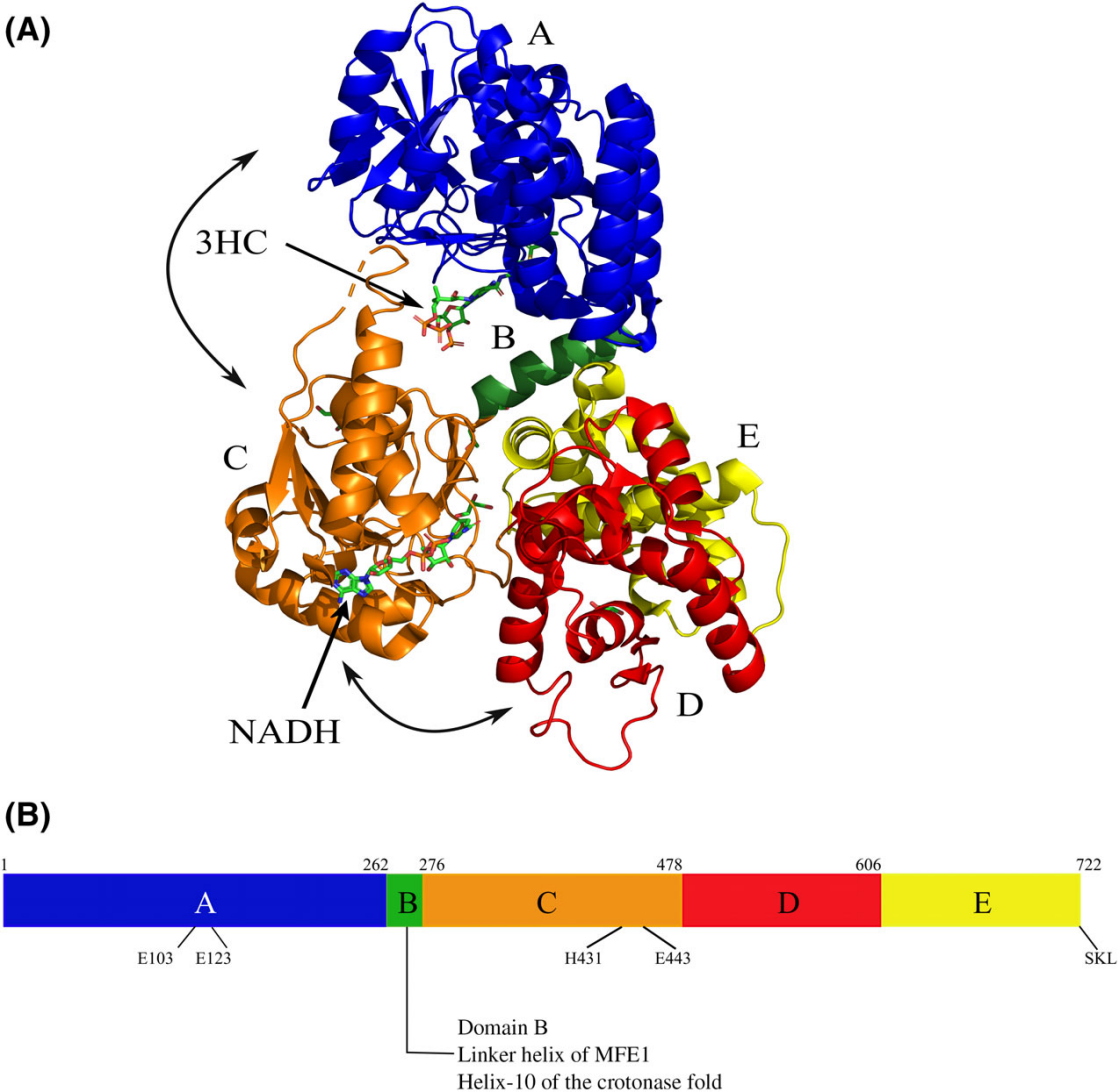
The fold of MFE1. (A) Color‐coded cartoon of the structure of MFE1 (PDB ID 6ZIC). The crotonase part is formed by domains A (blue) and B (green). The HAD part is formed by domains C (orange, the NAD binding domain) and D(red)/E(yellow). The ligand bound in the ECH active site (3HC) is 3*S*‐hydroxybutanoyl‐CoA. The ligand bound in the HAD active site is NADH. The curved arrows identify the two hinge motions, being the conformation of domain A with respect to the D/E‐domains and the conformation of domain C with respect to the D/E‐domains. (B) Schematic diagram, defining the domain structure of MFE1 and identifying the key catalytic residues, located in domain A (ECH active site) and domain C (HAD active site). The BCDE variant of RnMFE1 starts at residue 260. SKL is the C‐terminal peroxisomal targeting sequence.

The ECH active site framework is also known as the crotonase fold, and this fold is known to be the framework for active sites of a wide range of CoA‐dependent enzymes [7]. The best‐characterized monofunctional 2*E*‐enoyl‐CoA hydratase is the rat mitochondrial hydratase, also known as crotonase [8]. Two catalytic glutamates are important for the catalysis of the hydratase reaction, which in RnMFE1 are Glu103 and Glu123 (Fig. 1). Glu103 activates the active site water and Glu123 is important for exchanging a proton with the C2 atom of the substrate. Domain B of MFE1 corresponds to the C‐terminal helix H10 of the crotonase fold. This helix covers the ECH active site. In the MFE1 structure, the domain B/H10 helix also connects the ECH part (domain A) with the HAD part (domains C, D and E) and it is tightly interacting with domain E of the HAD part (Fig. 2). The structural studies of MFE1 have shown that domain A can adopt different positions with respect to domain B and domain E (of the HAD part), due to small conformational changes of the peptide region between domain A and domain B, as also visualized in Fig. 2 [9].

The HAD active site structure of MFE1 is similar as found in the homologous monofunctional mitochondrial enzymes [10, 11]. Human HAD (HsHAD) [8] is the best‐characterized monofunctional HAD enzyme. HsHAD is a dimeric NAD‐dependent 3*S*‐hydroxyacyl‐CoA dehydrogenase, in which each subunit (35 kDa) consists of an NAD binding domain and a dimerization domain (Fig. 3). The catalytic site is at the interface of the NAD binding domain and the dimerization domain. Similarly, the HAD part of MFE1 consists of the NAD binding domain (domain C) followed by domains D and E which both have the same topology as the dimerization domain of HsHAD and which interact tightly with each other (Fig. 3). Domain D completes the HAD active site (together with domain C). Domain E is the C‐terminal domain of MFE1 and this domain corresponds to the dimerization domain of the second chain of the dimeric HAD [5] (Fig. 3). The available crystal structures of RnMFE1 have captured structures in which domain C is either in an open or in a more closed conformation with respect to the D/E‐domains [9] (Fig. 2), depending on the hinge conformation of the connecting peptide between the C‐ and D‐domains of RnMFE1. Several crystal structures of HsHAD are also available, showing that the NAD binding domain (domain C in MFE1) adopts open and closed conformations, which correlates with the absence or presence of ligand(s) in the active site, respectively. In the absence of any ligand, the more open conformation is observed. The structure of a fully closed conformation of the ternary complex of HsHAD, in which both NAD^+^ as well as acetoacetyl‐CoA (AcAc‐CoA) are bound in the active site, has also been obtained (PDB ID 1F0Y) [12] (Table 1). This complex is a dead‐end complex as both the nucleotide (NAD^+^) as well as the substrate (AcAc‐CoA) are present in their oxidized form. The latter, fully closed, structure is important for understanding the HAD reaction mechanism, showing that in the HAD dehydrogenation reaction, a hydride is transferred from the C3 atom of 3*S*‐hydroxyacyl‐CoA to the C4 carbon atom of the nicotinamide group of NAD^+^, while a proton is transferred from the 3*S*‐hydroxy group to the side chain of His158. The side chain of His158 is anchored by its hydrogen bond interaction with a glutamate side chain (Glu170 in HsHAD, also of the NAD‐binding domain). In the HAD active site of RnMFE1, the important catalytic residue is His431, for exchanging a proton with the 3‐keto/3*S*‐hydroxy atom of the substrate (Fig. 1). The His431 side chain is anchored by the Glu443 side chain, corresponding to Glu170 of HsHAD.

**Fig. 3:**
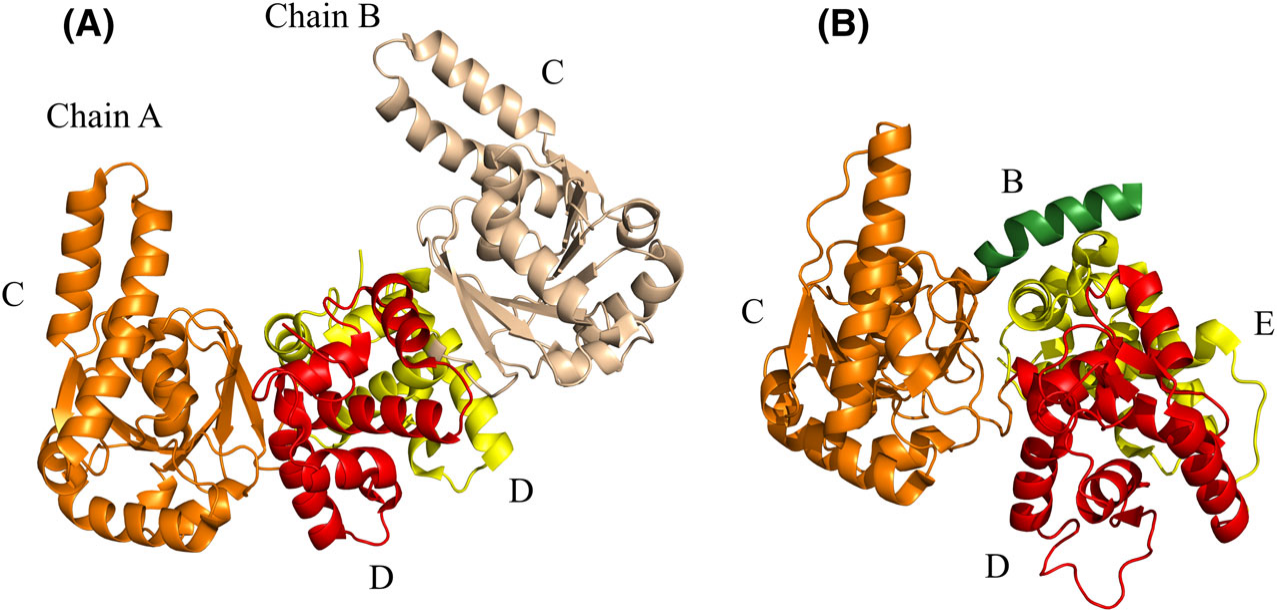
The domain structure of the HsHAD dimer and the BCDE variant of RnMFE1. (A) The structure of the HsHAD dimer (PDB ID 1F0Y). Chain A (orange, labeled C: NAD binding domain, red, labeled D: dimerization domain) is in the same view as used in Fig. 2. Chain B, shown in yellow color (dimerization domain, labeled D) and wheat color (NAD binding domain, labeled C) is the second chain of the HsHAD dimer. The dimer two‐fold axis is located between the two dimerization domains. (B) The fold of the BCDE variant (PDB ID 1ZCJ) (same view and color code as in Fig. 2). In this variant domain A is missing. Domain B/H10 (the linker helix) is in green, domain C in orange, domain D in red and domain E in yellow. Domain D of BCDE corresponds to the dimerization domain of chain A of HsHAD and domain E of BCDE corresponds to the dimerization domain of chain B of HsHAD.

**Table 1:** Characteristic distances concerning the hinge conformations of the RnMFE1, E123A, BCDE and HsHAD structures.

Structure: PDB ID‐chain^a^	Ligand(s) bound in HAD active site	Hinge conformation of domain C^b^	Thr306‐Ala524 distance^b^ (Å)	Hinge conformation of domain A^c^	Gly100‐Ala524 distance^c^ (Å)
RnMFE1					
6ZIC‐A	NADH	Closed	10.6	Open	43.9
6ZIC‐B	NADH	Open	12.8	Closed	42.1
6ZIB‐A	NADH	Closed	10.8	Open	44.0
6ZIB‐B	NADH	Open	12.8	Closed	42.9
5MGB‐A	NAD^+^	Closed	10.2	Open	43.1
5MGB‐B	NAD^+^	Open	12.8	Closed	41.5
3ZWC‐A	NADH	Closed	10.2	Open	43.5
3ZWA‐B	NADH	Open	12.9	Closed	41.8
E123A					
3ZWB‐A	Unliganded	Closed	9.9	Open	43.3
3ZWB‐B	Unliganded	Open	13.4	Closed	41.8
BCDE					
1ZCJ‐A	Unliganded	Open	12.8		
HsHAD	Ligand(s) bound in HAD active site	Hinge conformation of domain C^b^	Leu25‐Val253 distance^b^ (Å)		
1F0Y‐A	NAD^+^, AcAc‐CoA	Fully closed	8.3		
1F14‐A	Unliganded	Open	12.0		
1F17‐A	NADH	Open	11.5		
3HAD‐A	NAD^+^	Open	11.5		
1F12‐A	3*S*‐hydroxybutanoyl‐CoA	Open	10.8		

^a^ 6ZIC is the 3OHC4‐NADH structure. 6ZIB is the 3OHC4‐NAD^+^ structure. 5MGB refers to the structure of PDB ID 5MGB [9] from a crystal of RnMFE1 obtained by cocrystallization in the presence of 2 mm AcAc‐CoA and 2 mm NAD^+^. 3ZWC refers to the structure of PDB ID 3ZWC [6] from a crystal of RnMFE1 grown in the presence of CoA, washed and subsequently soaked in the presence of 0.2 mm 2*E*‐decenoyl‐CoA and 0.2 mm NADH. 3ZWB refers to the structure of PDB ID 3ZWB [6] from a crystal of the E123A variant, grown in the presence of 0.2 mm 2*E*‐hexenoyl‐CoA. 1ZCJ refers to the structure of PDB ID 1ZCJ [14] from a crystal of the BCDE variant. 1F0Y is the HsHAD structure of the ternary complex (PDB ID 1F0Y, [12]), which is the most closed structure of HsHAD, obtained by cocrystallization of HsHAD in the presence of 5 mm NAD^+^ and 10 mm AcAc‐CoA.

^b^ This distance defines the width of the cleft between domain C and domain D of the HAD fold, as visualized in Fig. S1. Thr306 is at the N‐terminus of the pyrophosphate binding helix (helix CH1) of the NAD binding domain (domain C) and Ala524 is the C‐terminal residue of helix DH3 of domain D. The latter residues correspond to Leu25 and Val253 in HsHAD, respectively.

^c^ This distance defines the hinge conformation of domain A with respect to the D/E‐domains, as visualized in Fig. S1. Gly100 is at the beginning of the active site helix H3 of domain A and Ala524 is the C‐terminal residue of helix DH3 of domain D.

RnMFE1 crystals are routinely obtained from a cocrystallization experiment in the presence of 2 mm CoA and these crystals have two RnMFE1 molecules in the asymmetric unit, molecule A and molecule B. In molecule A, the ECH A‐domain is in a more open conformation and the HAD part is in a more closed conformation, whereas in molecule B the ECH part is in a more closed conformation, but the HAD part is in a more open conformation. The ECH active sites of molecules A and B are complexed with CoA and the HAD active sites are unliganded in these RnMFE1 crystals.

Crystal soaking experiments using wild‐type RnMFE1 crystals with substrate, such as 2*E*‐hexenoyl‐CoA and 2*E*‐decenoyl‐CoA, have resulted in structures in which the ECH active site of molecules A and B are complexed with the product, 3*S*‐hydroxyacyl‐CoA [6], showing that at least one of the hydratase active sites is active in this crystal form. These 3*S*‐hydroxyacyl‐CoA product molecules are not bound in the HAD active sites of molecule A or B. However, crystallographic binding studies with 2*E*‐decenoyl‐CoA in the presence of NAD^+^ have shown ligand binding in the HAD active site of molecule A, which has been modeled as 3‐ketodecanoyl‐CoA [13], suggesting that the HAD active site of molecule A is competent for catalysis, being able to convert NAD^+^ and 3*S*‐hydroxydecanoyl‐CoA into NADH and 3‐ketodecanoyl‐CoA. The latter mode of binding has also been observed in crystallographic binding studies with 3‐ketodecanoyl‐CoA [13], and it is known that 3‐ketoacyl‐CoA molecules of various chain lengths are high‐affinity inhibitors of the monofunctional HAD enzymes [11]. From the studies of Barycki and Banaszak concerning the HsHAD conformational flexibility, it is proposed that only the fully closed HsHAD active site is able to catalyze efficiently the dehydrogenase reaction [12]. The characteristic distance of the fully closed HsHAD active site (Table 1, Fig. S1), as seen in the structure of the ternary HsHAD dead‐end complex (PDB ID 1F0Y), is 8.3 Å. The corresponding distance in the RnMFE1 structure is 10.2 Å (closed) in molecule A and 12.8 Å (open) in molecule B [9] (Table 1). This distance is a measure of the width of the cleft between domain C and domain D of the HAD part. In the fully closed HsHAD structure of its ternary complex, the nicotinamide and the 3‐keto moieties are tightly stacked, sandwiched between a methionine (Met26) and an asparagine (Asn208) side chain of domain C and the dimerization domain, respectively [12]. The conformational switch transforming molecule A into molecule B of the RnMFE1 crystal causes the HAD active site of molecule B to adopt a more open (inactive) conformation, whereas its hydratase active site adopts a more closed conformation (Table 1). At the moment, it is not clear if this conformational switch between these two RnMFE1 molecules is the result of crystal packing or if it is related to the intrinsic dynamical properties of this enzyme, or both.

Crystallographic studies have also been reported for two variants of RnMFE1 whose kinetic properties are characterized further in the studies reported here, being the E123A variant and the BCDE variant. In the E123A point mutation variant, the hydratase active site is inactive, as it lacks one of the catalytic glutamates, Glu123, being changed into an alanine, and indeed, in this case, 2*E*‐enoyl‐CoA is bound in the hydratase active site (without being hydrated), when its crystals are grown in the presence of the substrate 2*E*‐hexenoyl‐CoA (PDB ID 3ZWB) [6]. Other structural studies have provided the unliganded crystal structure of the BCDE variant (PDB ID 1ZCJ) [14, 15]. In this variant, domain A, critically important for the ECH activity, is missing. The structure of the BCDE variant is very similar to the structure of the HAD part of the full‐length RnMFE1, including its linker helix (domain B), which is not present in HsHAD (Fig. 3).

The studies reported here focus on comparing the dehydrogenase properties of RnMFE1 and HsHAD using as substrate *3S*‐hydroxybutanoyl‐CoA, which is converted into 3‐ketobutanoyl‐CoA (also referred to as AcAc‐CoA). In particular, it concerns steady state and pre‐steady state enzyme kinetic studies of the dehydrogenase activity of wild‐type RnMFE1, its point mutated E123A variant [6], its truncated BCDE variant [14], as well as of wild‐type HsHAD [12, 16]. The kinetic data show that RnMFE1 and its two variants are less efficient dehydrogenases than HsHAD and it is discussed that the difference of their catalytic properties correlates with the higher complexity of the RnMFE1 structure compared to HsHAD. In addition, the crystal structures of RnMFE1 complexed with, respectively, 3*S*‐hydroxybutanoyl‐CoA and AcAc‐CoA, only bound in the ECH active sites, show the hydrogen bond interactions of the 3*S*‐hydroxy and 3‐keto moieties of these ligands with the two catalytic glutamates of the ECH active site.

## Materials and methods

### Expression and purification of wild‐type RnMFE1

The RnMFE1 encoding cDNA, cloned into the pET15b (Novagen) plasmid at NdeI and BamHI sites with a N‐terminal (His)_6_‐tag, was available due to the previous studies of this enzyme [5]. In this construct 20 amino acid residues (Met‐Gly‐Ser‐Ser‐(His)_6_‐Ser‐Ser‐Gly‐Lys‐Val‐Pro‐Arg‐Gly‐Ser‐His) are added before the N‐terminal methionine of the RnMFE1 gene sequence. The recombinant protein was expressed by transforming this plasmid into BL21(DE3) pGro7 cells, such that the protein was co‐expressed with the GroEL and GroES chaperones encoded by the pGro7 plasmid (Takara Bio Inc., Shiga, Japan). The primary culture was grown overnight at 37 °C for which a single colony was inoculated into 10 mL of LB medium containing ampicillin (100 μg·mL^−1^) and chloramphenicol (20 μg·mL^−1^). The cells from the primary culture were used to grow the secondary culture in 1000 mL of M9ZB medium along with ampicillin (100 μg·mL^−1^), chloramphenicol (20 μg·mL^−1^), and 0.3 mg·mL^−1^ L‐arabinose to induce the expression of the chaperones. The culture was grown at 37 °C until A_600_ reached 0.8, and the expression was initiated by the addition of isopropyl‐β‐1‐thio‐d‐galactopyranoside (IPTG) to a final concentration of 0.1 mm, at 20 °C for overnight incubation. The cells were harvested by centrifugation. The cells were resuspended into the lysis buffer [20 mm potassium phosphate buffer, pH 7.4, 50 mm imidazole, 500 mm NaCl, 10% glycerol, 0.1% Triton X‐100, 100 μg·mL^−1^ lysozyme, 25 μg·mL^−1^ DNase, 25 μg·mL^−1^ RNase, 2 protease inhibitor tablets (Sigma‐Aldrich, St. Louis, MO, USA), 5 mm ATP, 5 mm MgCl_2_] and incubated for 30 min at room temperature. The cells were further lysed by sonication and were pelleted by centrifugation to separate the soluble fraction and cell debris. The supernatant was loaded onto a Ni‐NTA column (Qiagen, Hilden, Germany), pre‐equilibrated with the equilibration buffer (20 mm potassium phosphate buffer, pH 7.4, 500 mm NaCl). The protein was eluted with a linear gradient of 0–300 mm imidazole in equilibration buffer. The peak fractions of the protein were pooled and concentrated by using 30 kDa Amicon Centricon concentrators (Merck, Darmstadt, Germany). The concentrated protein solution was loaded onto a Superdex‐200 (GE Healthcare, Chicago, IL, USA) size exclusion chromatography column, pre‐equilibrated with the gelfiltration buffer [10 mm piperazine‐N,N′‐bis(2‐ethanesulfonic acid) (PIPES), pH 6.5, and 50 mm NaCl]. The latter buffer is also the storage buffer. The peak fractions were pooled and concentrated by using 30 kDa Centricon concentrators. The concentration of the protein was determined from absorption measurements at 280 nm with the NanoDrop 1000 spectrophotometer (ThermoFisher Scientific, Waltham, MA, USA), using the sequence‐based absorption coefficient provided by protparam (https://web.expasy.org/protparam). The protein solution was aliquoted into smaller fractions, flash frozen with liquid nitrogen and stored at −70 °C for further usage. The protein was confirmed to be pure by SDS/PAGE.

### Expression and purification of the E123A and the BCDE variants of RnMFE1

The E123A cDNA, cloned into the pET15b (Novagen) plasmid at NdeI and BamHI sites with a N‐terminal (His)_6_‐tag was available from previous studies [6]. This construct is the same as used for the wild‐type RnMFE1, except for the E123A point mutation. The expression, purification and storage protocols were the same as for wild‐type RnMFE1, as described above.

The BCDE encoding cDNA was also available from previous studies [14]. In these previous studies, the gene was cloned and expressed without a (His)_6_‐tag. In the current studies the BCDE gene was cloned into the pET15b (Novagen) plasmid at NdeI and BamHI sites with a N‐terminal (His)_6_‐tag, identical as used for the RnMFE1 and E123A constructs, but now preceding Ala260 of the ASGQ peptide of RnMFE1. In this construct, the N‐terminal end of the RnMFE1 part (Ala260) is the same as the N‐terminus of a proteolytic breakdown product (enzyme V) purified and characterized in studies of rat peroxisomal enzymes [17]. The recombinant protein was expressed by transforming the plasmid into BL21(DE3) pLysS cells, without the pGro7 plasmid. The primary culture was grown overnight at 37 °C, after which a single colony was inoculated into 10 mL of LB medium containing ampicillin (100 μg·mL^−1^) and chloramphenicol (20 μg·mL^−1^). The cells from this primary culture were used to grow the secondary culture containing 1000 mL of M9ZB medium along with ampicillin (100 μg·mL^−1^), and chloramphenicol (20 μg·mL^−1^). The culture was incubated at 37 °C until A_600_ reached 0.8, and the expression was initiated by the addition of IPTG to a final concentration of 0.1 mm, at 20 °C for overnight growth. The cells were harvested by centrifugation. The cells were then resuspended into the lysis buffer [50 mm 4‐(2‐hydroxyethyl)‐1‐piperazineethanesulfonic acid (HEPES), pH 7.6, 1 mm ethylenediaminetetraacetic acid (EDTA), 0.5 mm phenylmethylsulfonyl fluoride (PMSF), 0.5 mm benzamidine HCl, 0.5 mm reduced dithiothreitol (DTT), 100 μg·mL^−1^ lysozyme, 25 μg·mL^−1^ DNase, 25 μg·mL^−1^ RNase] and incubated for 30 min at room temperature. The cells were further lysed by sonication and were subsequently pelleted by centrifugation to separate the soluble fraction and cell debris. The supernatant was mixed with Ni‐NTA beads pre‐equilibrated with buffer (50 mm HEPES, pH 7.6, 1 mm EDTA). The protein was eluted with 300 mm imidazole in this equilibration buffer. The protein was concentrated by using 10 kDa Amicon Centricon concentrators (Merck). The concentrated protein was loaded onto the Superdex‐200 (GE Healthcare) size exclusion chromatography column, pre‐equilibrated with the gelfiltration buffer (100 mm HEPES, pH 7.5). This buffer is also the storage buffer. The peak fractions were pooled and concentrated by using 10 kDa Centricon concentrators. The concentration of the protein was determined from absorption measurements at 280 nm with the NanoDrop 1000 spectrophotometer (ThermoFisher Scientific), using the sequence‐based absorption coefficient provided by protparam (https://web.expasy.org/protparam). The protein solution was aliquoted into smaller fractions, flash frozen with liquid nitrogen and stored at −70 °C for further usage. The protein was confirmed to be pure by SDS/PAGE. The stability of the two RnMFE1 variants, as judged from the *T*_m_ values, calculated from their CD melting curves, is similar as the stability of wild‐type RnMFE1 (Table S1).

### Expression and purification of HsHAD

The human mitochondrial dehydrogenase encoding cDNA, cloned into the pET28a plasmid with a N‐terminal (His)_6_‐tag, was provided by Dr. Barycki, University of Nebraska, USA. The sequence of the plasmid was verified by sequencing in the Biocenter Oulu Sequencing Center. The recombinant protein was expressed by transforming the plasmid into BL21(DE3) pLysS cells. The primary culture was grown overnight at 37 °C, after which a single colony was inoculated into 10 mL of LB medium containing kanamycin (100 μg·mL^−1^) and chloramphenicol (20 μg·mL^−1^). The cells from this primary culture were used to grow the secondary culture containing 1000 mL of M9ZB medium along with kanamycin (100 μg·mL^−1^), and chloramphenicol (20 μg·mL^−1^). The culture was incubated at 37 °C until A_600_ reached 0.8, and the expression was initiated by the addition of IPTG to a final concentration of 0.1 mm, at 20 °C for overnight growth. The cells were harvested by centrifugation. The cells were then resuspended into the lysis buffer [20 mm tris(hydroxymethyl)‐aminomethane (Tris), pH 7.8, 300 mm NaCl, 5 mm imidazole, 100 μg·mL^−1^ lysozyme, 25 μg·mL^−1^ DNase, 25 μg·mL^−1^ RNase] and incubated for 30 min at room temperature. The cells were further lysed by sonication and pelleted by centrifugation to separate the soluble fraction and cell debris. The supernatant was mixed with Ni‐NTA beads pre‐equilibrated with buffer (20 mm Tris, pH 7.8, 300 mm NaCl, 20 mm imidazole). The protein was eluted with 250 mm imidazole in this equilibration buffer. The protein was concentrated by using 10 kDa Amicon Centricon concentrators (Merck). The concentrated protein solution was loaded onto the Superdex‐200 (GE Healthcare) size exclusion chromatography column, pre‐equilibrated by gelfiltration buffer (50 mm potassium phosphate, pH 7.2, 100 mm NaCl), which is also the storage buffer. The peak fractions were pooled and concentrated by using 10 kDa Centricon concentrators. The concentration of the protein was determined from absorption measurements at 280 nm with the NanoDrop 1000 spectrophotometer (ThermoFisher Scientific), using the sequence‐based absorption coefficient provided by protparam (https://web.expasy.org/protparam). The protein was aliquoted into smaller fractions, flash frozen with liquid nitrogen and stored at −70 °C for further usage. The protein was confirmed to be pure by SDS/PAGE.

### CD spectrophotometric and CD thermal shift experiments

Circular dichroism (CD) spectroscopy was performed using a Chirascan CD spectrophotometer (Applied Photophysics, Leatherhead, UK). The protein samples were diluted with milliQ water to a concentration of 0.1 mg·mL^−1^. CD data were collected between 260 and 190 nm at 22 °C using a 0.1 cm path‐length quartz cuvette. CD measurements were acquired every 1 nm with 0.5 s as an integration time and repeated three times with baseline correction. Data were processed using Chirascan Pro‐Data Viewer (Applied Photophysics), and CDNN (courtesy of and written by Dr. Gerald Böhm, 1997, Institut für Biotechnologie, Martin‐Luther Universität Halle‐Wittenberg, Germany, distributed by Applied Photophysics). The direct CD measurements (θ, mdeg) were converted into mean residue molar ellipticity (θ, MRME) by Pro‐Data Viewer. Thermal denaturation of the protein samples was monitored by measuring the CD spectra in the same setup with a temperature range from 22 to 90 °C at a rate of 1 °C·min^−1^ using a Peltier Temperature Control TC125 (Quantum Northwest, Liberty Lake, WA, USA). The CD data were recorded at every second °C. The *T*_m_ was calculated with the global3 software (Applied Photophysics) using a one‐transition model.

### The substrates 2*E*‐butenoyl‐CoA and 3*S*‐hydroxybutanoyl‐CoA

2*E*‐butenoyl‐CoA was purchased from Sigma‐Aldrich. NAD^+^, and NADH were also purchased from Sigma‐Aldrich. 3*S*‐hydroxybutanoyl‐CoA was synthesized from ethyl‐3*S*‐hydroxybutyrate (purchased from Merck, Darmstadt, Germany) by protecting the hydroxy group as tetrahydropyranylether, hydrolysis of the ester, followed by activation and coupling to CoA using the mixed anhydride method [18] and removal of the protection group. The identity and purity of the final product 3*S*‐hydroxybutanoyl‐CoA were checked by thin layer chromatography (TLC) and high‐resolution liquid‐chromatography‐mass‐spectrometry (LC–MS) analysis. Before use, the substrates were dissolved in 50 m m Tris buffer (pH 9.0). The concentration of the substrates in their stock solution was routinely determined by performing the Ellman's test [19, 20].

### Steady state Michaelis–Menten kinetics of the HAD dehydrogenase activity

The Michaelis–Menten enzyme kinetic measurements were carried out with the JASCO V660 spectrophotometer (JASCO Corporation, Tokyo, Japan) at 25 °C for 3 min in a 1 cm cuvette with a reaction volume of 0.5 mL. The initial rates were obtained by varying the substrate concentration after the optimal enzyme concentration had been determined. The reaction was initiated by adding the enzyme. The JASCO software spectramanager was used for carrying out the experiments and for the subsequent calculations. The initial, linear part of the progress curve was used to obtain the initial rates from which the *k*_cat_ and *K*_M_ values were determined. The values of *k*_cat_ and *K*_M_ are the averages of at least three measurements and their error bars are the standard deviations derived from the variation of these measurements.

The 3*S*‐hydroxyacyl‐CoA dehydrogenase activity was measured using 2.0 μg·mL^−1^ RnMFE1 (24 nm), 2.0 μg·mL^−1^ E123A (24 nm), 2.0 μg·mL^−1^ BCDE (40 nm) and 40 ng·mL^−1^ HsHAD (1.2 nm subunit) concentrations. The substrate concentration varied from 0.8 to 200 μm. In the dehydrogenase assay 3*S*‐hydroxyacyl‐CoA is converted into 3‐ketoacyl‐CoA in the presence of NAD^+^ as coenzyme. In this assay, the rates are measured by monitoring the absorption change at 340 nm due to the conversion of NAD^+^ to NADH. The assay buffer contained 50 mm Tris (pH 9.0), 50 mm KCl, and 50 μg·mL^−1^ BSA (bovine serum albumin), as well as 1 mm NAD^+^ [21], in agreement with the observation that in the dehydrogenase reaction the optimum pH is pH = 9 [11]. For calculating the initial rates from the absorption changes, an absorption coefficient of 6200 m ^−1^·cm^−1^ was used.

### Pre‐steady state kinetic (stopped‐flow) experiments

The stopped‐flow experiments were carried out with an SX20 stopped‐flow system from Applied Photophysics. Two types of experiments were performed, being (a) the determination of the *k*_on_, *k*_off_ rate constants of NAD^+^ and NADH and (b) the determination of *k*_chem_ for the dehydrogenase reaction in the forward direction. Both experiments were carried out using 50 mm Tris, pH 9.0, 50 mm KCl as the assay buffer, at room temperature. The signal was recorded by monitoring the change in fluorescence as a function of time. The fluorescence change is either due to a change in the intrinsic tryptophan fluorescence of the protein in case of the NAD^+^ binding experiment (excitation wavelength is 290 nm, using a cutoff filter of 320 nm) or due to a change of fluorescence of the cofactor in case of the NADH binding experiment and the *k*_chem_ experiment (excitation wavelength is 340 nm, using a cutoff filter of 420 nm). For each shot 100 μL from each reagent was used. Usually for each measurement, 5–10 traces were recorded and averaged. The averaged traces were analyzed using the pro‐data software (Applied Photophysics) using Eqn (1) for determination of the apparent rate constant, *k*_obs_, where F is the fluorescence signal, *A* is the signal amplitude, *t* is the time and *C* is the floating end point.

(1) $$F=A^{-k_{\mathrm{obs}}t}+C$$

### *k*_on_ and *k*_off_ rate constants of NAD ^+^ and NADH binding of RnMFE1, E123A, BCDE and HsHAD

In the pre‐steady state binding studies, the NAD^+^ and NADH concentrations were varied between 10 and 50 μm and the enzyme concentration was 1 or 2 μm. The obtained rate constants (*k*_obs_) with their errors were plotted against the different concentrations of the cofactor. The *k*_on_ and *k*_off_ rate constants and their errors were obtained using Eqn (2) as the LINFIT function of the simfit software (https://www.simfit.org.uk/).

(2) $$k_{\mathrm{obs}}=k_{\mathrm{on}}\left[\text{NADH}\right]+k_{\mathrm{off}}$$

### *k*_chem_ of the HAD dehydrogenase activity of RnMFE1, E123A, BCDE and HsHAD

In the *k*_chem_ stopped‐flow studies, the enzyme was pre‐equilibrated with 1 mm NAD^+^. *k*_obs_ was determined, by varying the 3*S*‐hydroxybutanoyl‐CoA or 2*E*‐butenoyl‐CoA concentration between 1 and 50 μm, while keeping the concentrations of the pre‐equilibrated enzyme and NAD^+^ constant. The obtained rate constants (*k*_obs_) and their errors were subsequently plotted against the different concentrations of the substrate. *k*_chem_ of the reaction and *K*_D_ of the substrate and their errors were obtained using QNFIT of the simfit software (https://www.simfit.org.uk/), using Eqn (3).

(3) $$k_{\mathrm{obs}}=\frac{k_{\text{chem}}\;\left[R‐\mathrm{OH}\right]}{K_{d}\left(R‐\mathrm{OH}\right)+\left[R‐\mathrm{OH}\right]}+C$$

### Crystallization, crystal treatment, data collection, and structure refinement

RnMFE1 crystals were obtained by the sitting drop, vapor diffusion method, using a solution of 8 mg·mL^−1^ protein dissolved in 10 mm PIPES, pH 6.5, and 50 mm NaCl, that was supplemented with 2 mm CoA and incubated for 30 min at room temperature, before setting up the crystallization experiments. The crystallization screen was formulated using the TECAN Freedom EVO‐100 pipetting robot. Using the Mosquito nanodispenser (TTP Labtech, Melbourn, UK), a sitting drop plate (flat bottom Greiner 609830, CrystalQuick™ Plus LBR, Jena Bioscience, Jena, Germany) was set up, where 1 μL of protein solution was mixed with 1 μL of crystallization well solution, and using 100 μL of reservoir solution. The plates were incubated at room temperature. The plates were imaged at regular time intervals using a Formulatrix RI54 imager and the results were monitored using the icebear software [22]. The crystallization well solution was a 75–125 mm 2‐(N‐morpholino)ethanesulfonic acid (MES) buffer, pH 6.0, containing also between 125 and 175 mm ammonium sulfate, and between 13% and 18% (w/v) polyethylene glycol (PEG) 4000. Crystals were obtained in about 3–4 days and allowed to grow for at least 2 weeks. Subsequently, crystals of this plate were used for two different crystal treatment protocols, as summarized in Table 2, being carried out at room temperature and resulting in the 3OHC4‐NADH and 3OHC4‐NAD^+^ data sets. These data sets were collected at the ESRF (Grenoble, France), using beam line ID23‐EH1.

**Table 2:** Crystallization, crystal treatment protocols and data collection of the 3OHC4‐NADH and the 3OHC4‐NAD^+^ structures.

Data set	3OHC4‐NADH	3OHC4‐NAD^+^
Crystal growth		
Protein buffer (1 μL)	8 mg·mL^−1^ dissolved in 10 mm PIPES, pH 6.5, and 50 mm NaCl, that was supplemented with 2 mm CoA and incubated for 30 min at room temperature	8 mg·mL^−1^ dissolved in 10 mm PIPES, pH 6.5, and 50 mm NaCl, that was supplemented with 2 mm CoA and incubated for 30 min at room temperature
Well solution (1 μL)	125 mm MES, pH 6.0; 18% w/v PEG4000; 175 mm ammonium sulfate	100 mm MES, pH 6.0; 16% w/v PEG4000; 150 mm ammonium sulfate
Crystal treatment		
Crystal washing and soaking solution (time, composition)	The crystal was washed overnight with the well solution and subsequently soaked in well solution supplemented with 0.2 mm 3*S*‐hydroxybutanoyl‐CoA + 2 mm NADH for 2 h	The crystal was washed overnight with the well solution and subsequently soaked in well solution supplemented with 0.2 mm 3*S*‐hydroxybutanoyl‐CoA + 2 mm NAD^+^ for 2 h
Cryo‐cooling (time, composition)	The crystal was quickly (few seconds) moved through well solution, supplemented with 0.2 mm 3*S*‐hydroxybutanoyl‐CoA + 2 mm NADH and diluted with glycerol (final concentration 20%), before being cryo‐cooled in liquid nitrogen	The crystal was quickly (few seconds) moved through well solution, supplemented with 0.2 mm 3*S*‐hydroxybutanoyl‐CoA + 2 mm NAD^+^ and diluted with glycerol (final concentration 20%), before being cryo‐cooled in liquid nitrogen
Sample name in IceBear [22]	RnMFE1_94kkB06d1c1	RnMFE1_94kkA10d1c1
Data collection		
Beam line	ESRF ID23‐EH1	ESRF ID23‐EH1
Detector	DECTRIS PILATUS 6 m	DECTRIS PILATUS 6 m
Wavelength (Å)	0.972957	0.972957
Temperature (K)	100	100
PDB ID	6ZIC	6ZIB

The 3OHC4‐NADH data set was processed at 2.2 Å resolution using xia2‐dials [23] and subsequently scaled and merged using aimless [24]. The crystal structure was solved by molecular replacement using phaser [25]. Molecule A of the RnMFE1 structure (PDB ID 5OMO) was used as the search model. Molecule A was split into three parts, being domain A, domains B and C and domains D/E. Two copies of the different parts were obtained as a solution of the molecular replacement. The correct orientation and position of the parts of the two molecules of the asymmetric unit were obtained in COOT [26] using the symmetry operations. The COOT graphical program was subsequently used to manually improve the model and to build the water structure and the ligands. The structure was refined using refmac5 [27] of the ccp4i2 package [28]. Toward the end of the refinement, TLS refinement was used to improve the structure further. The TLS groups were chosen manually, following the domain structure of MFE1, such that four groups were identified, being domains A, B, C and D/E. The quality of the structure was monitored by using the validation tools of COOT and by inspecting the PDB validation report. The structure was refined to a final *R*_work_ of 20.0% and *R*_free_ of 23.3% (Table 3). The ECH active sites of molecule A and molecule B are complexed with 3*S*‐hydroxybutanoyl‐CoA (Fig. S2) and both HAD active sites are liganded only with NADH (Fig. S2).

**Table 3:** Data processing statistics and refinement statistics of the 3OHC4‐NADH and the 3OHC4‐NAD^+^ structures.

Data set	3OHC4‐NADH	3OHC4‐NAD^+^
Space group	P2_1_ 2_1_2_1_	P2_1_ 2_1_2_1_
*a*, *b*, *c* (Å)	65.64, 127.09, 227.10	65.94, 127.39, 228.17
α, β, γ (°)	90.00, 90.00, 90.00	90.00, 90.00, 90.00
Data processing software	XIA2‐DIALS, AIMLESS	XDSAPP, AIMLESS
Resolution (Å)^a^	65.1–2.20 (2.24–2.20)	48.9–2.70 (2.78–2.70)
R_pim_	0.036 (0.386)	0.060 (0.406)
CC_1/2_	0.998 (0.715)	0.995 (0.691)
I/σ(I)	11.7 (2.1)	8.3 (2.0)
Completeness (%)	99.6 (100.0)	95.5 (96.4)
Redundancy	7.6	7.8
Observed reflections	734 763 (37 089)	397 907 (36 328)
Unique reflections	96 924 (4732)	51 199 (4422)
Wilson B‐factor (Å^2^)	38.6	52.6
Refinement statistics		
Resolution	65.1–2.20	48.9–2.70
Number of used reflections	91 900	48 331
*R*_work_ (%)	20.0	23.2
*R*_free_ (%)	23.3	27.3
Total number of atoms	11 611	11 379
Number of waters	255	67
Average B‐factor:		
Molecule A/B (Å^2^)	47.5/62.9	62.3/83.4
Active site ligands (Å^2^)^b^	114.1 (3HC‐A‐ECH), 70.4 (3HC‐B‐ECH), 56.1 (NAI‐A‐HAD), 56.6 (NAI‐B‐HAD)	107.2 (CAA‐A‐ECH), 79.9 (CAA‐B‐ECH), 86.7 (NAI‐A‐HAD), 102.5 (NAI‐B‐HAD)
Waters (Å^2^)	43.4	47.0
RMS deviation:		
Bond lengths (Å)	0.0071	0.0037
Bond angles (°)	1.6	1.1
Ramachandran plot:^c^		
Favored region (%)	96.3	95.7
Allowed region (%)	3.2	3.9
Outlier region (%)	0.5	0.4
PDB ID	6ZIC	6ZIB

^a^ The numbers in parentheses refer to the resolution range of the outer shell.

^b^ 3HC, CAA, NAI are the PDB ligand codes for, respectively, 3*S*‐hydroxybutanoyl‐CoA, AcAc‐CoA, and NADH.

^c^ As calculated by MolProbity [48].

The 3OHC4‐NAD^+^ data set was processed at a resolution of 2.7 Å with XDSAPP [29, 30] and scaled and merged using AIMLESS. The structure was solved as described above for the 3OHC4‐NADH data set. Toward the end of the refinement of this structure, TLS refinement was used to improve the structure further, as described above. The quality of the structure was monitored by using the validation tools of COOT and by inspecting the PDB validation report. The structure was refined to a final *R*_work_ of 23.2% and *R*_free_ of 27.3% (Table 3). For this structure, the crystal was soaked with 3*S*‐hydroxybutanoyl‐CoA and NAD^+^ and it is expected that the HAD active site catalyzes the conversion of 3*S*‐hydroxybutanoyl‐CoA and NAD^+^ into AcAc‐CoA and NADH, respectively [13]. The electron density map for the acyl tail of the ligand bound in the ECH active site is better defined in the 3OHC4‐NAD^+^ structure (Fig. S3) than in the 3OHC4‐NADH structure (Fig. S2), which is in line with the presence of AcAc‐CoA bound in the former active site (as it is expected to be a tighter binder in the ECH active site than 3*S*‐hydroxybutanoyl‐CoA [31]). Its mode of binding is the same as observed in the structure of a crystal that was obtained by a different protocol, being from a cocrystallization experiment of RnMFE1 in the presence of 2 mm AcAc‐CoA and 2 mm NAD^+^ (Fig. S4) (PDB ID 5MGB) [9]. In the latter structure, AcAc‐CoA is bound in both ECH active sites, whereas both HAD active sites are complexed with only NAD^+^. For the 3OHC4‐NAD^+^ and 3OHC4‐NADH structures, it is observed that the electron density for the nicotinamide moiety of the nucleotide (Figs S2 and S3) is well defined in both structures, being better than observed in the 5MGB structure of RnMFE1 complexed with NAD^+^ in the HAD active site. In the 5MGB structure, the nicotinamide moiety is in poor density, like also seen in the structures of HsHAD complexed with NAD^+^ [12]. In the 3OHC4‐NAD^+^ structure and in the 3OHC4‐NADH structure the electron density of the nicotinamide moiety is similar as in previous structures of RnMFE1‐NADH complexes (for example 3ZWC) [6]. Therefore, in the final 3OHC4‐NAD^+^ structure both ECH active sites have a bound AcAc‐CoA molecule, and both HAD active sites have a bound NADH molecule.

### Structure analysis

The structures were compared with the structures of PDB ID 5MGB (2.8 Å resolution) [9], PDB ID 3ZWC (2.3 Å resolution) [6], and PDB ID 3ZWB (3.1 Å resolution) [6]. The 5MGB structure had been obtained from a crystal of RnMFE1 obtained by cocrystallization in the presence of 2 mm AcAc‐CoA and 2 mm NAD^+^ and this structure has AcAc‐CoA bound in the ECH active site and NAD^+^ in the HAD active site of both molecules of the asymmetric unit. The 3ZWC structure had been obtained from a crystal of RnMFE1, grown in the presence of CoA, washed and subsequently soaked in the presence of 0.2 mm 2*E*‐decenoyl‐CoA and 0.2 mm NADH. This structure has 3*S*‐hydroxydecanoyl‐CoA bound in the ECH active sites and NADH in the HAD active sites. The 3ZWB structure had been obtained from a crystal obtained by cocrystallization of E123A in the presence of 0.2 mm 2*E*‐hexenoyl‐CoA, which is bound only in the ECH active sites. For structure comparisons, the structures were superimposed using the SSM protocol [32], as implemented in COOT. For the superposition figures the structures were superimposed by the LSQ option of COOT, using domain A. The figures of the structures were made with pymol (Schrödinger LLC, New York, NY, USA).

## Results and Discussion

### Steady state and pre‐steady state kinetic experiments with RnMFE1 and HsHAD

The steady state enzyme kinetic data provided the *k*_cat_ and *K*_M_ values from the Michaelis–Menten curves of the dehydrogenase activities (Fig. S5, Table 4). Table 4 lists the comparison of the *k*_cat_ and *K*_M_ values for the short chain 2*E*‐butenoyl‐CoA and 3*S*‐hydroxybutanoyl‐CoA substrates of wild‐type RnMFE1, its variants and wild‐type HsHAD. *k*_cat_ for 2*E*‐butenoyl‐CoA (requiring both the hydratase as well as the dehydrogenase reaction) and 3*S*‐hydroxybutanoyl‐CoA (requiring only the HAD active site) for RnMFE1 are the same. The kinetic constants for the forward dehydrogenation reaction for the substrate 3*S*‐hydroxybutanoyl‐CoA for wild‐type RnMFE1 and its E123A and BCDE variants are also in the same range (between 1.6 and 2.4 s^−1^), being much lower as measured for HsHAD (42 s^−1^). The *K*_M_ value of 2*E*‐butenoyl‐CoA for RnMFE1 is 3.6 μm, and for 3*S*‐hydroxybutanoyl‐CoA it varies between 5.2 and 11 μm for RnMFE1 and its variants. The *K*_M_ of 3*S*‐hydroxybutanoyl‐CoA for HsHAD is 19 μm.

**Table 4:** The Michaelis–Menten kinetic constants of the dehydrogenase activities of RnMFE1, E123A, BCDE and HsHAD for the 2*E*‐butenoyl‐CoA and 3*S*‐hydroxybutanoyl‐CoA substrates.

Enzyme	Substrate	*k*_cat_ (s^−1^)	*K*_M_ (μm)
RnMFE1^a^	2*E*‐butenoyl‐CoA	1.7 ± 0.1	3.6 ± 1
RnMFE1^b^	3*S*‐hydroxybutanoyl‐CoA	1.9 ± 0.3	11 ± 2
E123A^b^	3*S*‐hydroxybutanoyl‐CoA	2.4 ± 0.1	10 ± 0.6
BCDE^b^	3*S*‐hydroxybutanoyl‐CoA	1.6 ± 0.1	5.2 ± 0.9
HsHAD^b^	3*S*‐hydroxybutanoyl‐CoA	42 ± 2	19 ± 0.9

^a^ Ref. [9].

^b^ Experimental details are in the Materials and methods section.

**Fig. 4:**
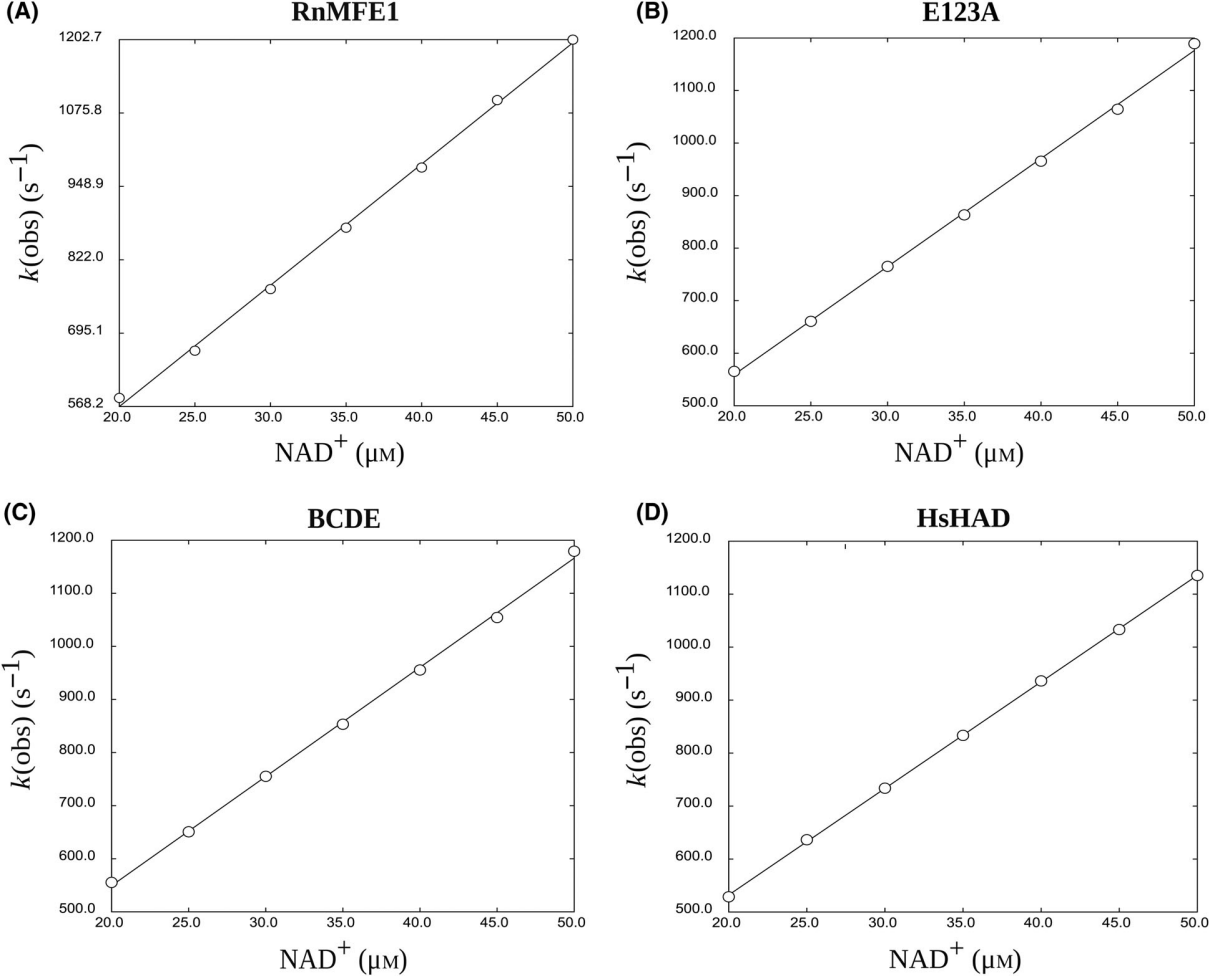
Determination of *k*_on_ and *k*_off_ of NAD^+^ binding by plotting *k*_obs_ as a function of NAD^+^ concentration. (A) Binding of NAD^+^ to RnMFE1 (2 μm). (B) Binding of NAD^+^ to E123A (2 μm). (C) Binding of NAD^+^ to BCDE (2 μm). (D) Binding of NAD^+^ to HsHAD (2 μm, subunit concentration). *k*_on_ and *k*_off_ are obtained by curve fitting using Eqn (2) and listed in Table 5.

**Fig. 5:**
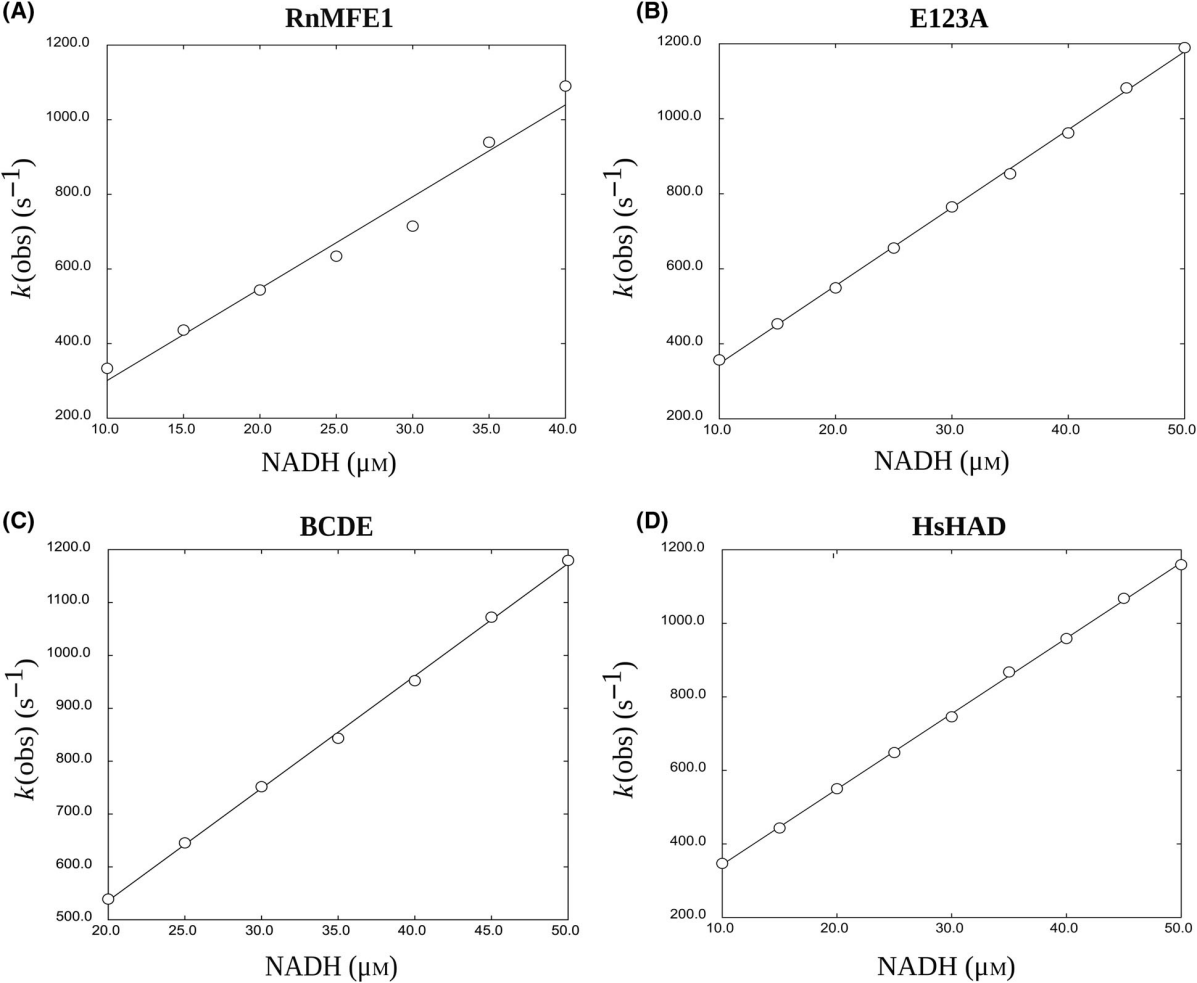
Determination of *k*_on_ and *k*_off_ of NADH binding by plotting *k*_obs_ as a function of NADH concentration. (A) Binding of NADH to RnMFE1 (1 μm). (B) Binding of NADH to E123A (2 μm). (C) Binding of NADH to BCDE (2 μm). (D) Binding of NADH to HsHAD (1 μm, subunit concentration). *k*_on_ and *k*_off_ are obtained by curve fitting using Eqn (2) and listed in Table 5.

Two types of stopped‐flow experiments were carried out: (a) the determination of *k*_on_ and *k*_off_ for the binding of NAD^+^ and NADH to the HAD active sites as well as (b) the determination of the *k*_chem_ of the dehydrogenation reaction, being the oxidation of 3*S*‐hydroxybutanoyl‐CoA to AcAc‐CoA by measuring the rate of formation of NADH by the HAD active site. The binding experiments (Figs 4 and 5, Table 5) show that the *k*_on_ and *k*_off_ values (for NAD^+^ and NADH) of RnMFE1 and HsHAD vary between 20 and 22 s^−1^·μm ^−1^ for *k*_on_ and between 110 and 160 s^−1^ for *k*_off_. The *K*_D_ (which corresponds to the ratio of *k*_off_/*k*_on_) varies between 5.0 and 8.0 μm, showing that the unliganded active site of RnMFE1 (and its two variants) and of HsHAD have very similar, high, affinity for NAD^+^ and NADH. Within the cell NAD^+^ is in much excess of NADH and the concentration of NAD^+^ is reported to be around 100 μm or higher in the cytosol, as well as in the mitochondria and peroxisomes [33, 34, 35] suggesting that in the cell RnMFE1 and HsHAD are complexed with NAD^+^ before subsequently forming the ternary complex with the 3*S*‐hydroxyacyl‐CoA substrate.

**Table 5:** Pre‐steady state binding studies with RnMFE1, E123A, BCDE and HsHAD for determining *k*_on_, *k*_off_ for NAD^+^ and NADH and their *K*_D_ values, calculated from the ratio of *k*_off_ and *k*_on_ ^a^.

Enzyme	Coenzyme	*k*_on_ (s^−1^ μm ^−1^)	*k*_off_ (s^−1^)	*K*_D_ (μm)
RnMFE1	NAD^+^	21 ± 8	160 ± 0.2	7.6
E123A	NAD^+^	20 ± 0.1	160 ± 4	8.0
BCDE	NAD^+^	20 ± 0.1	150 ± 4	7.5
HsHAD	NAD^+^	20 ± 0.1	120 ± 3	6.0
RnMFE1	NADH	22 ± 0.9	110 ± 20	5.0
E123A	NADH	20 ± 0.2	150 ± 3	7.5
BCDE	NADH	21 ± 0.1	120 ± 4	5.7
HsHAD	NADH	20 ± 0.1	140 ± 2	7.0

^a^ Experimental details are in Figs 4 and 5 and in the Materials and methods section.

### The *k*_chem_ rate constants are much larger than the *k*_cat_ rate constants for RnMFE1 and HsHAD

**Fig. 6:**
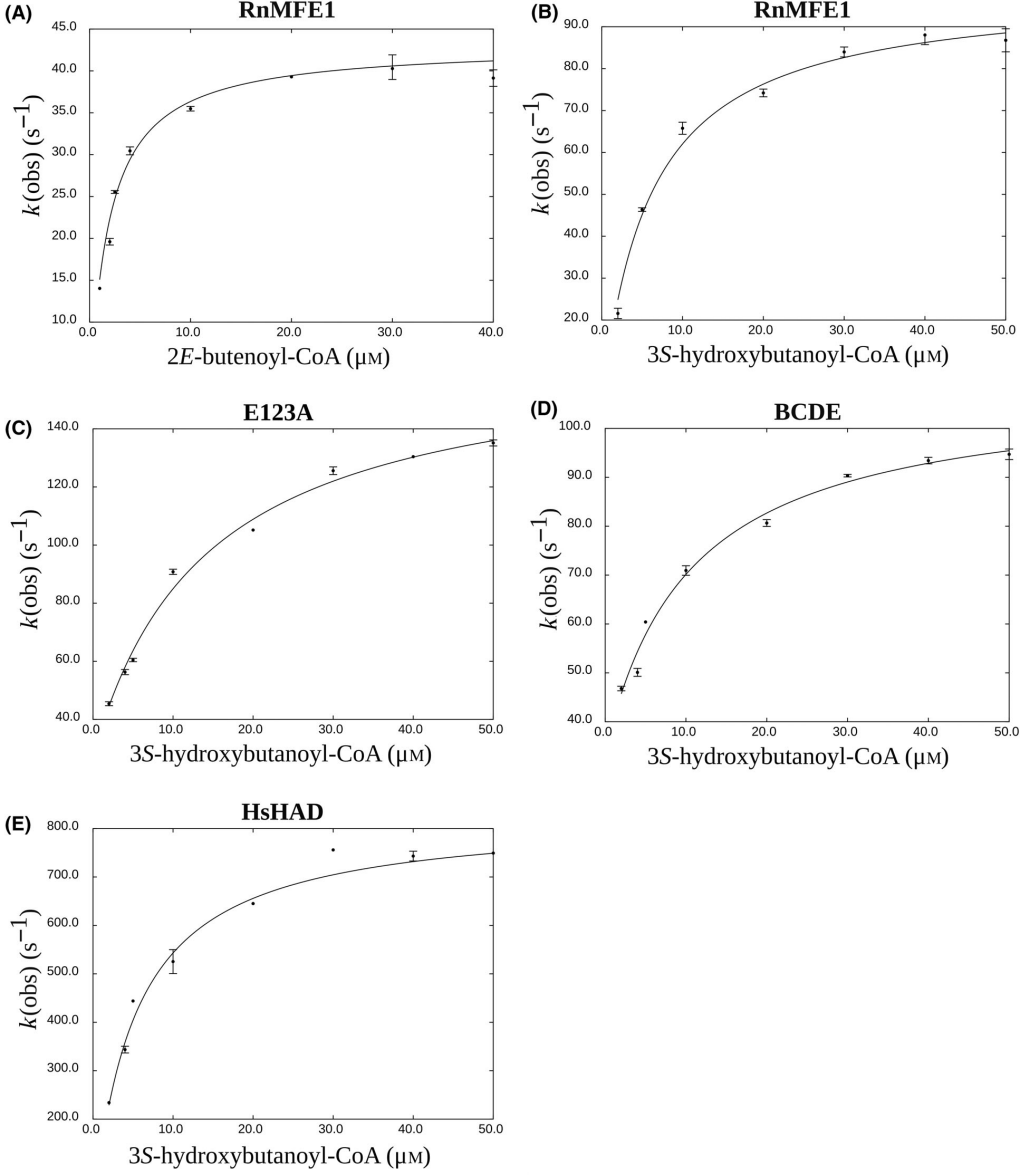
Determination of *k*_chem_ of the dehydrogenation reaction by plotting *k*_obs_ as a function of substrate concentration. (A) RnMFE1 (1 μm) with the 2*E*‐butenoyl‐CoA substrate. (B) RnMFE1 (1 μm) with the 3*S*‐hydroxybutanoyl‐CoA substrate. (C) E123A (1 μm) with the 3*S*‐hydroxybutanoyl‐CoA substrate. (D) BCDE (1 μm) with the *3S*‐hydroxybutanoyl‐CoA substrate. (E) HsHAD (1 μm, subunit concentration) with the 3*S*‐hydroxybutanoyl‐CoA substrate. *k*_chem_ and *K*_D_ are obtained by curve fitting using Eqn (3) and listed in Table 6. The error bars visualize the range of the observed *k*_obs_ values.

The pre‐steady state *k*_chem_ measurements of the dehydrogenation reaction, concerning the formation of the AcAc‐CoA product, were done with the substrates 2*E*‐butenoyl‐CoA and 3*S*‐hydroxybutanoyl‐CoA, using enzyme that was pre‐equilibrated with 1 mm NAD^+^. The formation of NADH was monitored by fluorescence measurements (measuring the fluorescence of the formed NADH) to obtain the *k*_obs_ values as a function of substrate concentration (Fig. 6) by which *k*_chem_ and *K*_D_ of the substrates 2*E*‐butenoyl‐CoA and 3*S*‐hydroxybutanoyl‐CoA were obtained (Table 6). For the assay with 2*E*‐butenoyl‐CoA the substrate for the dehydrogenation reaction is generated by the hydratase active site. The *k*_chem_ rate constant for this substrate for RnMFE1 is 43 s^−1^. For the substrate 3*S*‐hydroxybutanoyl‐CoA, *k*_chem_ rate constants were obtained for RnMFE1 (99 s^−1^), E123A (140 s^−1^), BCDE (75 s^−1^) and HsHAD (830 s^−1^). The obtained *K*_D_ values for the substrates of these five experiments are 1.9 μm for 2*E*‐butenoyl‐CoA for RnMFE1 and between 5.9 and 14 μm for 3*S*‐hydryxbutanoyl‐CoA for RnMFE1, E123A, BCDE and HsHAD.

**Table 6:** The *k*_chem_ and *K*_D_ values of RnMFE1, E123A, BCDE and HsHAD for the 2*E*‐butenoyl‐CoA and 3*S*‐hydroxybutanoyl‐CoA substrates in the presence of 1 mm NAD^+^ ^a^.

Enzyme	Substrate	*k*_chem_ (s^−1^)	*K*_D_ (μm)
RnMFE1	2*E*‐butenoyl‐CoA	43 ± 1	1.9 ± 0.1
RnMFE1	3*S*‐hydroxybutanoyl‐CoA	99 ± 2	5.9 ± 0.4
E123A	3*S*‐hydroxybutanoyl‐CoA	140 ± 6	14 ± 2
BCDE	3*S*‐hydroxybutanoyl‐CoA	75 ± 3	11 ± 2
HsHAD	3*S*‐hydroxybutanoyl‐CoA	830 ± 20	5.2 ± 0.3

^a^ Experimental details of these pre‐steady state measurements of NADH formation are in Fig. 6 and in the Materials and methods section.

The *k*_cat_ and *k*_chem_ rate constants for 2*E*‐butenoyl‐CoA for the hydratase/dehydrogenation reactions by RnMFE1 are 1.7 s^−1^ (Table 4) and 43 s^−1^ (Table 6), which are in the same range as for 3*S*‐hydroxybutanoyl‐CoA, respectively 1.9 and 99 s^−1^. This shows that neither the rate of the hydratase reaction nor the rate of the transfer of the hydrated intermediate between the ECH active site and the HAD active site is a rate‐limiting step for the formation of AcAc‐CoA from 2*E*‐butenoyl‐CoA.

The *k*_cat_ and *k*_chem_ rate constants have also been reported in the classical studies on the monofunctional horse liver alcohol dehydrogenase (hlADH). This enzyme is also NAD dependent, but in this enzyme, the substrate alcohol is not conjugated to CoA [36]. For this enzyme, the *K*_M_ values of the substrate are in the mm range (instead of the μm range as observed for RnMFE1 and HsHAD, Table 4). This enzyme has a completely different fold, and its enzymatic activity is dependent on a Zn^2+^ ion bound in the active site. Like in HsHAD, also in hlADH a domain closure movement is an essential feature of the catalytic cycle [37, 38]. In the fully closed form of hlADH the distance between the C4 carbon atom of the nicotinamide ring of NAD^+^ and the carbon atom of the substrate is about 3.4 Å and this short distance is required for hydride transfer [39]. The reaction mechanism is proposed to be ordered such that in the forward direction the nucleotide cofactor, NAD^+^, binds first and subsequently the substrate is bound. The *k*_cat_ and *k*_chem_ rate constants for hlADH for the substrate ethanol are, respectively, 3.5 and 490 s^−1^ [36].

**Fig. 7:**
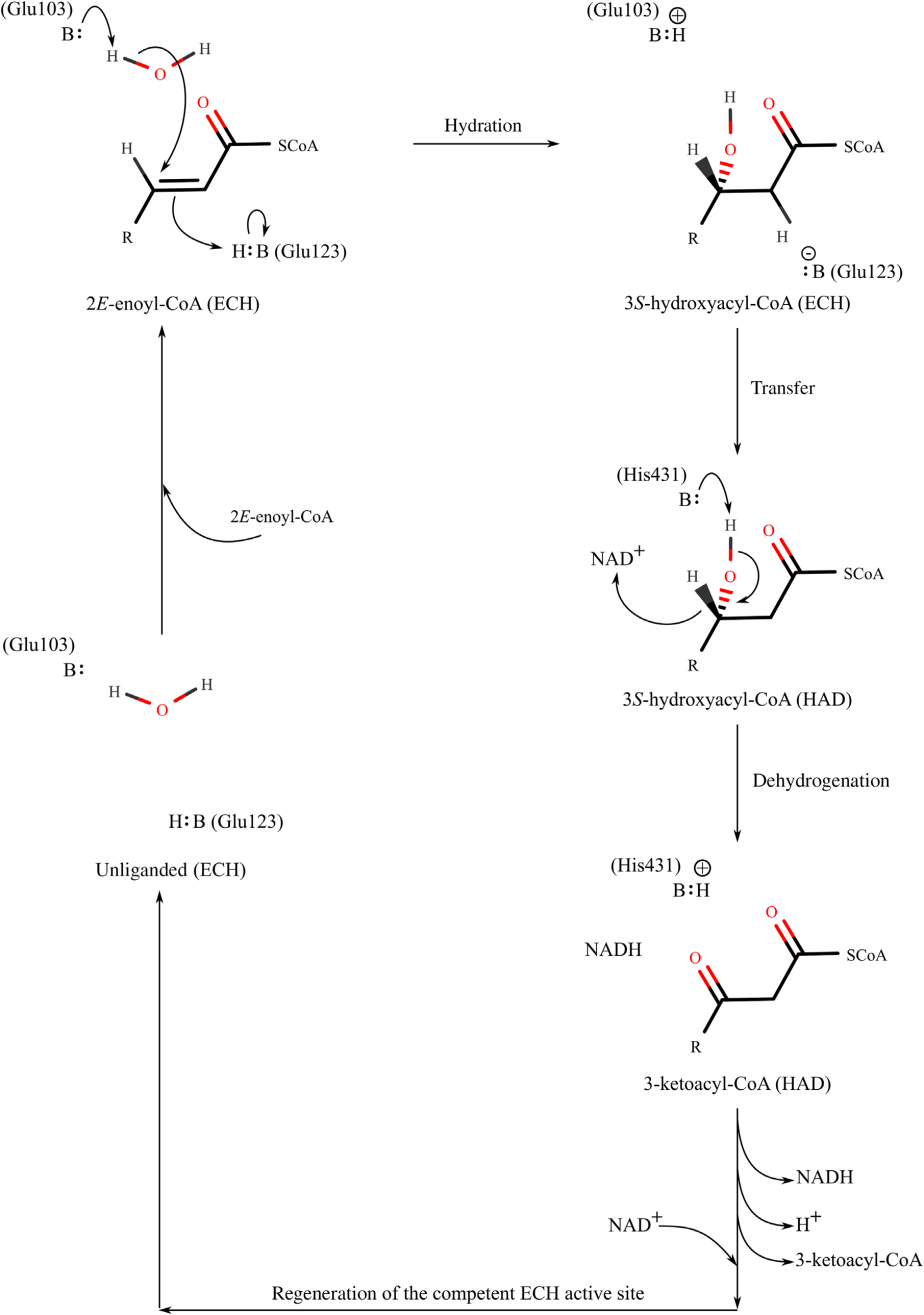
Schematic representation of the reaction cycle, as catalyzed by RnMFE1. The hydration reaction is catalyzed by the ECH active site and the dehydrogenation reaction is catalyzed by the HAD active site. The kinetic data suggest that the rate limiting step of the overall reaction cycle, starting from the 2*E*‐enoyl‐CoA hydratase reaction, concerns a step after formation of NADH by the dehydrogenation reaction of the HAD active site.

For both RnMFE1 as well as HsHAD, *k*_chem_ (Table 6) is much larger than *k*_cat_ (Table 4), respectively 50 and 20 fold faster for the substrate 3*S*‐hydroxybutanoyl‐CoA, which shows that, in both enzymes, hydride transfer (characterized by *k*_chem_) is not the rate‐limiting step of the overall reaction (characterized by *k*_cat_). Such a large difference in *k*_cat_ and *k*_chem_ has also been observed for hlADH, in which case the *k*_chem_ is approximately 100 fold faster than *k*_cat_ [36]. This is a common phenomenon in NAD‐dependent dehydrogenases, and it is understood that the dissociation of NADH or, more general, the regeneration of the catalytic site, is the rate‐limiting step of the catalytic cycle of these enzymes [36]. Apparently, some steps of the regeneration of the active sites, after the formation of NADH, have lower rates (Fig. 7). This can concern the dissociation of the products, but can also be due to conformational differences or different protonation states between product‐bound and substrate‐bound active sites. For example, the protonation state of the glutamates in the ECH active site and the histidine in the HAD active site of the enzyme product and the enzyme substrate complexes are different (Figs 1 and 7). The rate of proton transfer between the active site and bulk water has been studied for hlADH, and the results suggest that in this active site a water mediated proton relay system between the catalytic site and bulk water could play a role in the regeneration step of its reaction mechanism [36]. For hlADH it is proposed that conformational changes, for example related to the dissociation of the reduced nucleotide, are rate limiting [36]. Also, in other enzymes conformational changes during the catalytic cycle are known to be the rate limiting step of the catalytic cycle [40].

### RnMFE1 is a slower enzyme than HsHAD

The *k*_cat_ values (Table 4) and *k*_chem_ values (Table 6) for the RnMFE1 (and its E123A and BCDE variants) catalyzed dehydrogenation step are, respectively, approximately 20 and 8 fold lower as compared to the corresponding rate constants of the HsHAD catalyzed dehydrogenation reaction for the substrate 3*S*‐hydroxybutanoyl‐CoA. The sequence identity between the HAD part of RnMFE1 and HsHAD is 33% [5]. A key structural difference between these two enzymes concerns the presence of the linker helix (domain B, between domain A and domain C, Fig. 2) in MFE1, which tightly interacts with domain E of the HAD part of MFE1. The linker helix is therefore a physical connection between the D/E‐domains and domain C, which is absent in HsHAD. This helix is also present in the E123A and BCDE variants of RnMFE1 (Fig. 3), for which the dehydrogenase kinetic properties are very similar to RnMFE1 (Tables 4, 5, 6). HsHAD studies have shown that the movement of domain C with respect to domain D is essential for its catalytic properties [12]. Only in the fully closed conformation of HsHAD, the nicotinamide ring and the 3*S*‐hydroxy/3‐keto moieties interact tightly with each other, such that the distance between the C4 carbon of the nicotinamide moiety and the C3 carbon of the substrate is 3.5 Å, allowing for efficient catalysis of the hydride transfer [12, 39]. As discussed above, for hlADH the conformational switch related to the dissociation of the NADH from the closed form of the ternary complex of hlADH is proposed to be the rate limiting step in the overall dehydrogenase reaction catalyzed by this dehydrogenase [36]. It is known that the dynamical properties of enzymes are important for their catalytic properties and that these dynamical properties are influenced by structural features far away from the catalytic site [41, 42, 43, 44]. Therefore, it can be inferred that in MFE1 the dynamical properties of domain C with respect to domain D will be different from HsHAD, due to the presence of the linker helix, which will effect the catalytic properties of its HAD active site, being shaped by its C‐ and D‐domains. Therefore, it seems well possible, that the lower catalytic rate constants for the dehydrogenase activity of RnMFE1 and its variants are related to the structural differences between RnMFE1 and HsHAD. In this respect, it can also be noted that HsHAD is a dimer and the interactions between its two dimerization domains are different from the interactions between the corresponding D‐ and E‐domains of MFE1 (Fig. 3), which could also effect the conformational flexibility properties of its active site and therefore its kinetic properties.

### The mode of binding of 3*S*‐hydroxybutanoyl‐CoA and acetoacetyl‐CoA in the ECH active site of RnMFE1

**Fig. 8:**
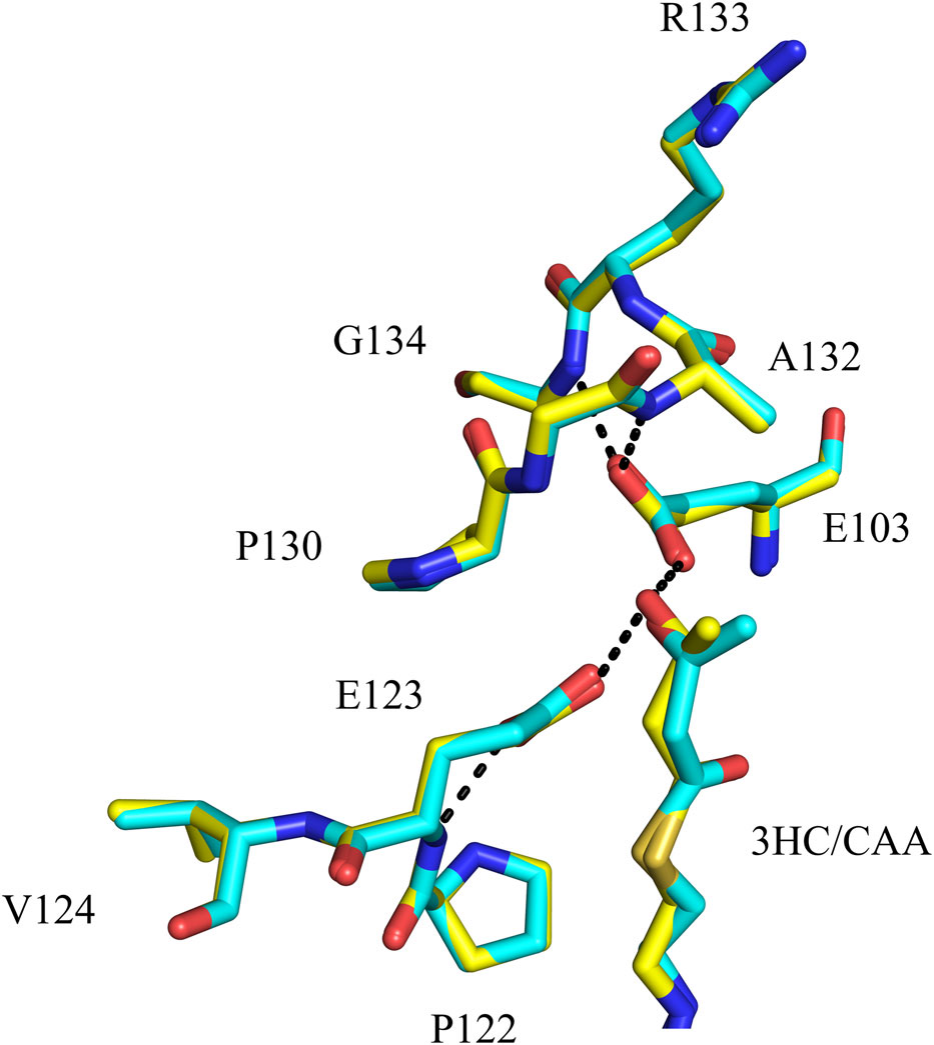
The conformation of the side chains of the catalytic glutamates, Glu103 and Glu123, of the hydratase active site of RnMFE1 is the same in the 3OHC4‐NAD^+^ and 3OHC4‐NADH structures. The ECH active site of the 3OHC4‐NAD^+^ structure (PDB ID 6ZIB, molecule A, complexed with AcAc‐CoA) is in cyan color and the active site of the 3OHC4‐NADH structure (PDB ID 6ZIC, molecule A, complexed with 3*S*‐hydroxybutanoyl‐CoA) is shown in yellow color. The hydrogen bond anchoring interactions of the side chains of Glu123 and Glu103 with, respectively the Pro122‐Glu123‐Val124 region [with N(Glu123)] and the Pro130‐Gly131‐Ala132‐Arg133‐Gly134 region [with N(Ala132) and N(Gly134)] are shown by dotted lines. Also highlighted by dotted lines are the hydrogen bond interactions between the catalytic glutamates and the 3*S*‐hydroxy oxygen atom of 3*S*‐hydroxybutanoyl‐CoA. The labels 3HC and CAA identify 3*S*‐hydroxybutanoyl‐CoA (yellow) and AcAc‐CoA (cyan), respectively.

In the two new RnMFE1 structures the HAD active site in molecule A is more closed, whereas in molecule B the HAD active site is more open (Table 1), like in the previously reported crystal structures of RnMFE1. In the 3OHC4‐NADH structure 3*S*‐hydroxybutanoyl‐CoA is bound in the ECH active sites of both molecules and in the 3OHC4‐NAD^+^ structure AcAc‐CoA is bound in the ECH active sites of both molecules, as described in the Materials and methods section. The HAD active sites of both molecules of both structures are complexed with NADH and not with 3*S*‐butanoyl‐CoA or AcAc‐CoA, which is in line with previous observations [6, 9]. Although the HAD active site of the crystallized MFE1 can convert 3*S*‐hydroxybutanoyl‐CoA into AcAc‐CoA [13], its affinity for 3*S*‐hydroxybutanoyl‐CoA and AcAc‐CoA is apparently low, which correlates with the notion that the conformation of its C‐domain is more open in this crystal form than in the fully closed conformation of the ternary complex of HsHAD, in both molecules of the asymmetric unit of both structures (Table 1). However, these binding studies do provide insight into the mode of interaction of 3*S*‐hydroxybutanoyl‐CoA and AcAc‐CoA with the catalytic glutamates (Glu103 and Glu123) in the ECH active site. In both structures, the main chain and side chain conformations of these residues are well defined by the electron density map, and there are no structural differences when comparing the conformations of the catalytic glutamates of these two active sites (Fig. 8). One of the carboxylate side chain oxygen atoms of both glutamates is anchored via hydrogen bonds to the main chain. The oxygen atom of the 3*S*‐hydroxy moiety as well as the 3‐keto moiety interacts via hydrogen bonds with the other carboxylate side chain oxygen atom of these glutamates (Fig. 8). The reaction mechanism of the hydration reaction (Fig. 1) suggests that in the 3*S*‐hydroxybutanoyl‐CoA product complex Glu103 will be protonated and Glu123 will be deprotonated, suggesting that the proton of the hydrogen bond between Glu123 and the product is provided by the proton of its 3*S*‐hydroxy moiety. This proton is absent in AcAc‐CoA, but due to keto‐enol tautomerization, AcAc‐CoA can exist in several forms in solution (Fig. 1C) [45, 46, 47], being not only the 3‐keto form, but it can occur also as its enol form, and as its deprotonated enolate form. The complex captured in the AcAc‐CoA structure concerns either an active site where Glu123 is deprotonated, being complexed with the enol form of AcAc‐CoA, or Glu123 is protonated, being complexed with the enolate form (Fig. 1C) or it is a mixture of both. Spectroscopic assays with the bovine mitochondrial monofunctional homolog [31] suggest that the enolate form is present in this complex, having a very high affinity (*K*_D_ is 1.7 μm) for this enzyme.

## Conclusion

The kinetic data show that the *K*_M_ values (Table 4) and the *K*_D_ values (Table 6) of the 3*S*‐hydroxybutanoyl‐CoA substrate for RnMFE1 and HsHAD are in the same range, between 5.2 and 19 μm. Likewise, the *K*_D_ values of NAD^+^ and NADH (Table 5) for RnMFE1 and HsHAD are in the same range, varying between 5.0 and 8.0 μm. Also, for both enzymes it is found that the rate limiting step of the overall reaction, when using as substrate 3*S*‐hydroxybutanoyl‐CoA, is the regeneration of the active site after the hydride transfer. However, the *k*_cat_ and *k*_chem_ rate constants of RnMFE1 (and its E123A and BCDE variants) are different from the corresponding values of HsHAD, being approximately 10 fold lower. The lower value for *k*_chem_ of the dehydrogenation reaction of RnMFE1 as compared to HsHAD suggests that in RnMFE1 the interactions in the active site are less favorable for stabilizing the high energy transition state geometry of the reaction cycle. Maybe the fully closed state, as observed in the structure of the HsHAD ternary complex [12], occurs with lower probability in RnMFE1, due to geometry constraints imposed by the presence of its linker helix (present in RnMFE1 and its two variants, but not present in HsHAD, Fig. 3), which interacts with domain E of the HAD part, but which is also physically connected to domain C. Alternatively, in the RnMFE1 ternary complex the ground state is stabilized better than in the HsHAD ternary complex. In any case, an RnMFE1 crystal structure in which domain C is in a fully closed conformation (as seen in the structure of the ternary HsHAD complex) has not yet been captured. The lower rate constant of the overall reaction of RnMFE1 (and its E123A and BCDE variants), *k*_cat_, could also be due to the presence of the MFE1 linker helix, possibly imposing conformational restraints on the dynamical properties of MFE1 and therefore effecting its catalytic properties. Further experimental and biocomputational studies have been initiated to understand better the interplay between the structural and dynamical properties of MFE1 and its enzyme kinetic properties.

## Conflict of interest

The authors declare no conflict of interest.

## Author contributions

SS, T‐RK, MW, RKW designed the project. WS provided the essential reagent. SS carried out the experiments. SS, T‐RK, MW, RKW analyzed the data. SS, T‐RK, MW and RKW prepared the manuscript with contributions from WS. MW and RKW provided the funding.

## Supporting information


**Fig. S1.** Visualization of the Cα‐Cα distances which are listed in Table 1.
**Fig. S2.** 2Fo‐Fc omit maps of the 3OHC4‐NADH structure (PDB ID 6ZIC) of (A) the bound 3*S*‐hydroxybutanoyl‐CoA (ECH active site, contour level is 0.7 sigma) and (B) the bound NADH (HAD active site, contour level is 1.0 sigma) of chain A.
**Fig. S3.** 2Fo‐Fc omit maps of the 3OHC4‐NAD^+^ structure (PDB ID 6ZIB) of (A) the bound AcAc‐CoA (ECH active site, contour level is 1.0 sigma) and (B) the bound NADH (HAD active site, contour level is 1.0 sigma) of chain A.
**Fig. S4.** The geometry of the hydratase active site of the 3OHC4‐NAD^+^ structure (obtained from a crystal soaked with 0.2 mM 3*S*‐hydroxybutanoyl‐CoA and 2 mM NAD^+^) is the same as observed in the structure obtained by cocrystallization in the presence of 2 mM AcAc‐CoA and 2 mM NAD^+^ (PDB ID 5MGB).
**Fig. S5.** Steady state kinetics Michaelis–Menten graph concerning the dehydrogenase reaction catalyzed by RnMFE1 for the substrate 3*S*‐hydroxybutanoyl‐CoA.
**Table S1.** The T_m_ values of RnMFE1 and its two variants as determined from CD melting curves.

## Data Availability

The atomic coordinates and structure factors of the models described in this manuscript were deposited into the Protein Data Bank with accession codes: 6ZIC (the 3OHC4‐NADH structure) and 6ZIB (the 3OHC4‐NAD^+^ structure).
