# Supplementary material for: Structural enzymology studies with the substrate 3S‐hydroxybutanoyl‐CoA: bifunctional MFE1 is a less efficient dehydrogenase than monofunctional HAD
Source: FEBS Open Bio. 2024 Mar 8;14(4):655–74. doi: 10.1002/2211-5463.13786 (PMC10988713; doi:10.1002/2211-5463.13786)
Supplement: Supplementary file 1 — Fig. S1. Visualization of the Cα‐Cα distances which are listed in Table 1. Fig. S2. 2Fo‐Fc omit maps of the 3OHC4‐NADH structure (PDB ID 6ZIC) of (A) the bound 3S‐hydroxybutanoyl‐CoA (ECH active site, contour level is 0.7 sigma) and (B) the bound NADH (HAD active site, contour level is 1.0 sigma) of chain A. Fig. S3. 2Fo‐Fc omit maps of the 3OHC4‐NAD+ structure (PDB ID 6ZIB) of (A) the bound AcAc‐CoA (ECH active site, contour level is 1.0 sigma) and (B) the bound NADH (HAD active site, contour level is 1.0 sigma) of chain A. Fig. S4. The geometry of the hydratase active site of the 3OHC4‐NAD+ structure (obtained from a crystal soaked with 0.2 mM 3S‐hydroxybutanoyl‐CoA and 2 mM NAD+) is the same as observed in the structure obtained by cocrystallization in the presence of 2 mM AcAc‐CoA and 2 mM NAD+ (PDB ID 5MGB). Fig. S5. Steady state kinetics Michaelis–Menten graph concerning the dehydrogenase reaction catalyzed by RnMFE1 for the substrate 3S‐hydroxybutanoyl‐CoA. Table S1. The Tm values of RnMFE1 and its two variants as determined from CD melting curves. [file FEB4-14-655-s001.docx]

**Supporting Information**

**Structural enzymology studies with the substrate 3*S*-hydroxybutanoyl-CoA: bifunctional MFE1 is a less efficient dehydrogenase than monofunctional HAD**

**Shruthi Sridhar^1^, Tiila-Riikka Kiema^2^, Werner Schmitz^3^, Mikael Widersten^4^, Rik K Wierenga^1*^**

^1^Faculty of Biochemistry and Molecular Medicine, University of Oulu, PO Box 5400, Oulu FI-90014, Finland

^2^Biocenter Oulu, University of Oulu, PO Box 5000, Oulu FI-90014, Finland

^3^Theodor Boveri Institute of Biosciences (Biocenter), University of Würzburg, Würzburg, Germany

^4^Department of Chemistry – BMC, Uppsala University, SE-751 23 Uppsala, Sweden

*correspondence E-mail: rik.wierenga@oulu.fi

**Table S1.** The T_m_ values of RnMFE1 and its two variants as determined from CD melting curves.

**Fig. S1.** Visualization of the Cα-Cα distances which are listed in **Table 1**.

**Fig. S2.** 2Fo-Fc omit maps of the 3OHC4-NADH structure (PDB ID 6ZIC) of (A) the bound 3*S*-hydroxybutanoyl-CoA (ECH active site, contour level is 0.7 sigma) and (B) the bound NADH (HAD active site, contour level is 1.0 sigma) of chain A.

**Fig. S3.** 2Fo-Fc omit maps of the 3OHC4-NAD^+^ structure (PDB ID 6ZIB) of (A) the bound AcAc-CoA (ECH active site, contour level is 1.0 sigma) and (B) the bound NADH (HAD active site, contour level is 1.0 sigma) of chain A.

**Fig. S4.** The geometry of the hydratase active site of the 3OHC4-NAD^+^ structure (obtained from a crystal soaked with 0.2 mM 3*S*-hydroxybutanoyl-CoA and 2 mM NAD^+^) is the same as observed in the structure obtained by cocrystallization in the presence of 2 mM AcAc-CoA and 2 mM NAD^+^ (PDB ID 5MGB).

**Fig. S5.** Steady state kinetics Michaelis-Menten graph concerning the dehydrogenase reaction catalyzed by RnMFE1 for the substrate 3*S*-hydroxybutanoyl-CoA.

**Table S1.** The T_m_ values of RnMFE1 and its two variants as determined from CD melting curves.

| Protein | T_m_ (°C) |
| --- | --- |
| RnMFE1 | 44.9 |
| E123A | 43.8 |
| BCDE | 41.6 |


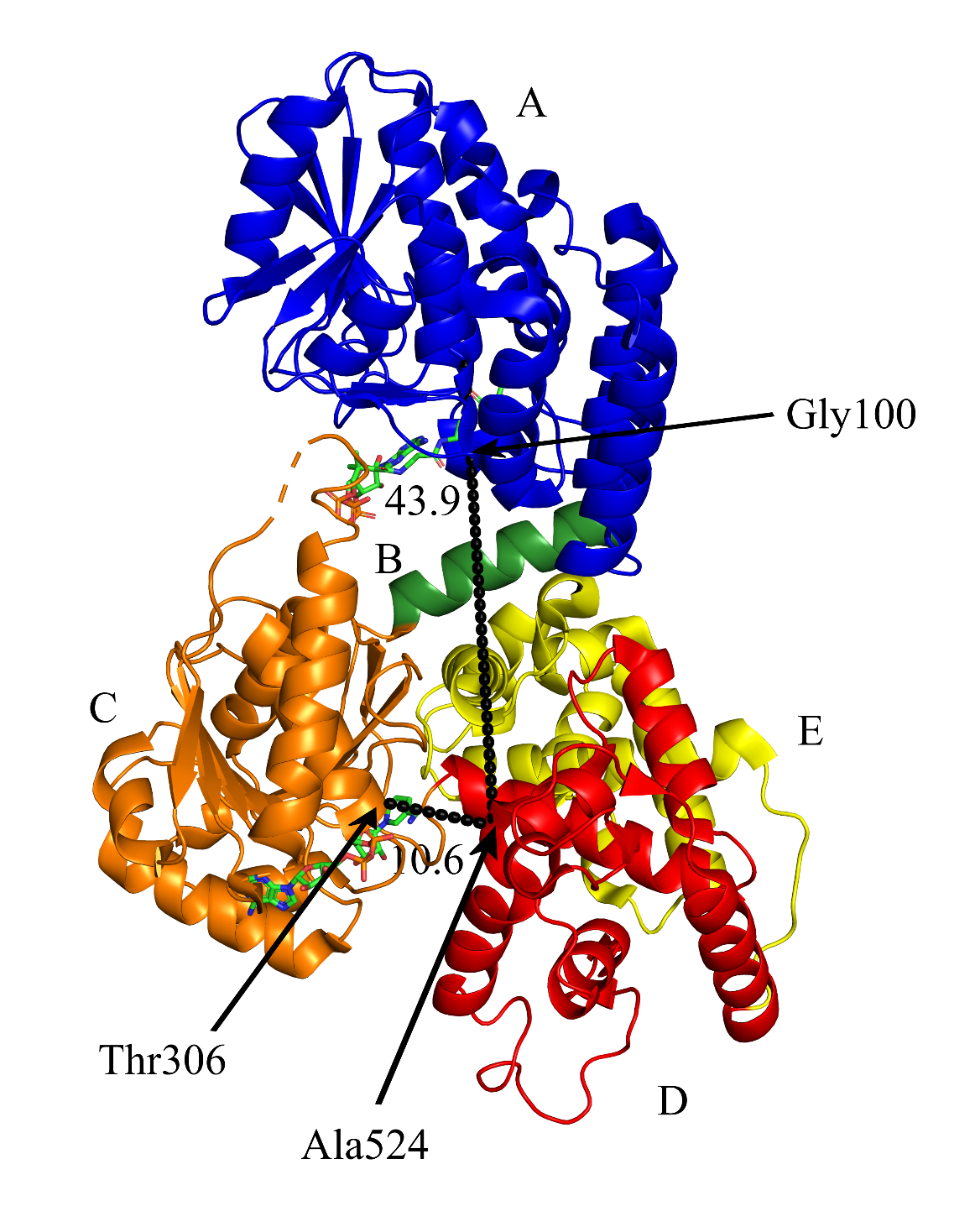


**Fig. S1.** **Visualization of the Cα-Cα distances which are listed in Table 1**. The distance between the Cα atoms of Gly100 (ECH active site) and Ala524 (domain D) characterizes the hinge conformation of domain A with respect to the D/E-domains. The distance between the Cα atoms of Thr306 (HAD active site) and Ala524 characterizes the hinge conformation of domain C with respect to the D/E-domains. The listed distances concern molecule A of the 3OHC4-NADH structure (PDB ID 6ZIC).

**
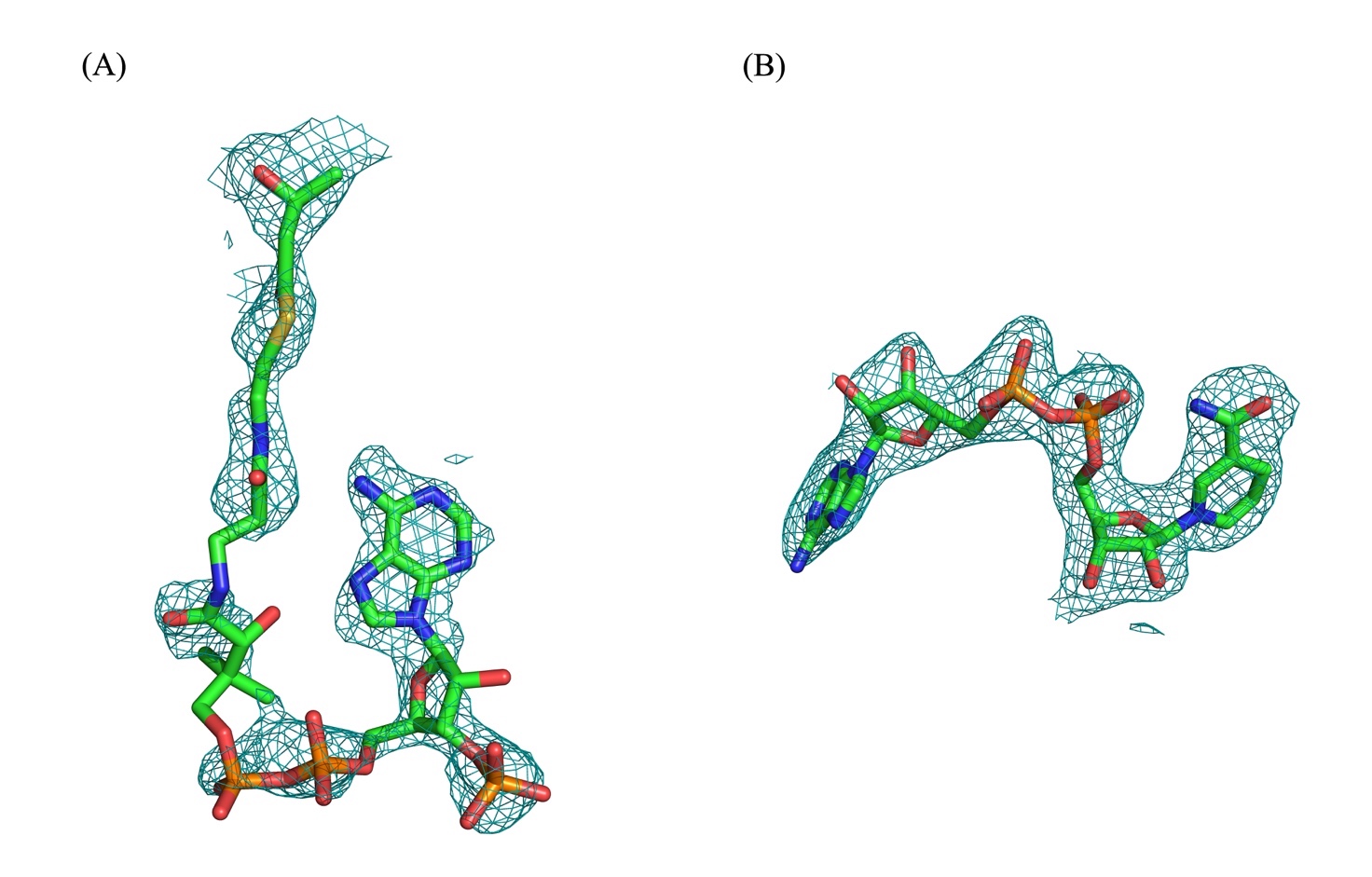
**

**Fig. S2. 2Fo-Fc omit maps of the 3OHC4-NADH structure (PDB ID 6ZIC) of (A) the bound 3*S*-hydroxybutanoyl-CoA (ECH active site, contour level is 0.7 sigma) and (B) the bound NADH (HAD active site, contour level is 1.0 sigma) of chain A.** The 2Fo-Fc omit maps were calculated using as a model the structure in which the shown ligand was removed from the model.

**
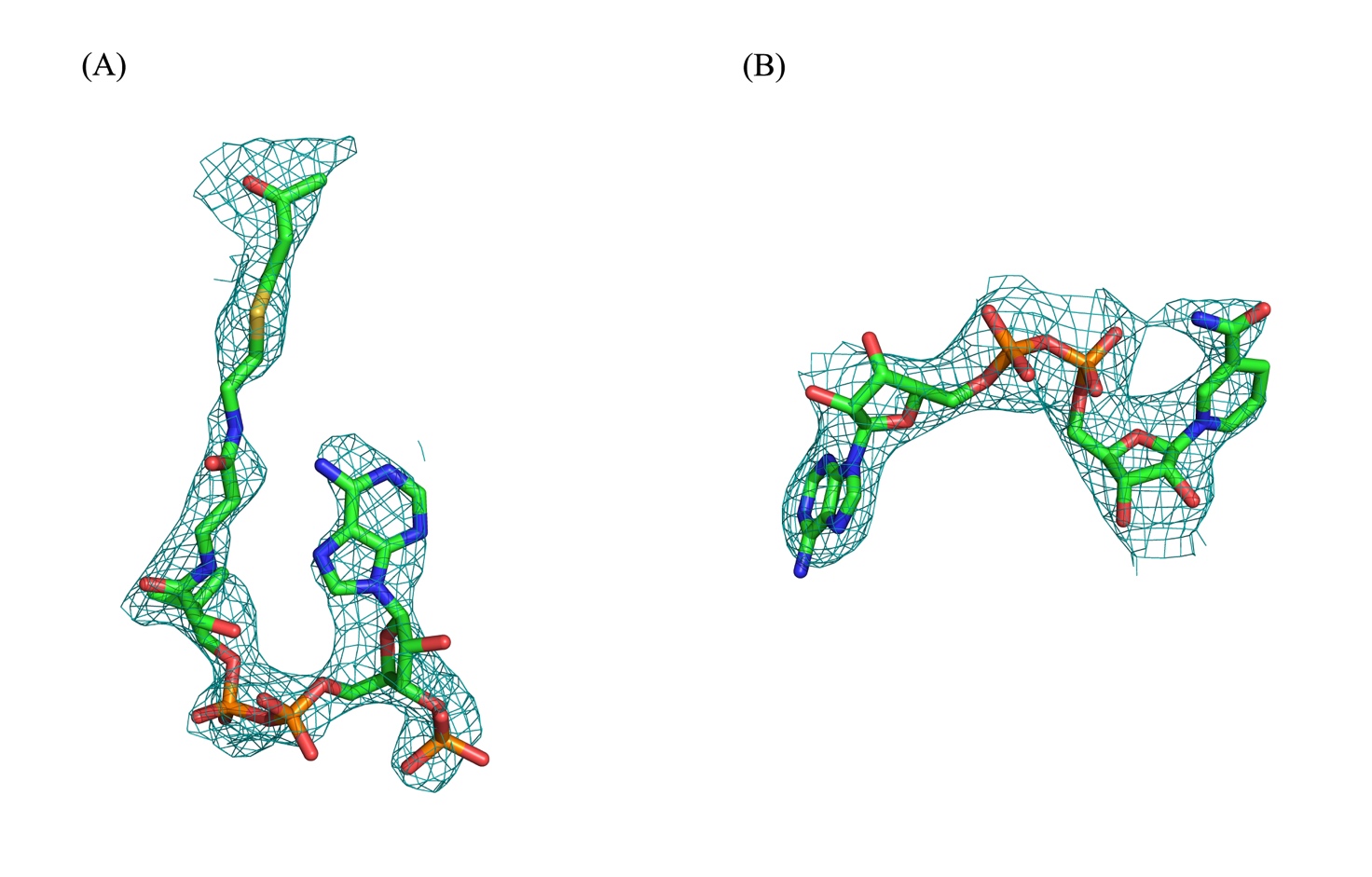
**

**Fig. S3. 2Fo-Fc omit maps of the 3OHC4-NAD^+^ structure (PDB ID 6ZIB) of (A) the bound AcAc-CoA (ECH active site, contour level is 1.0 sigma) and (B) the bound NADH (HAD active site, contour level is 1.0 sigma) of chain A.** The 2Fo-Fc omit maps were calculated using as a model the structure in which the shown ligand was removed from the model.


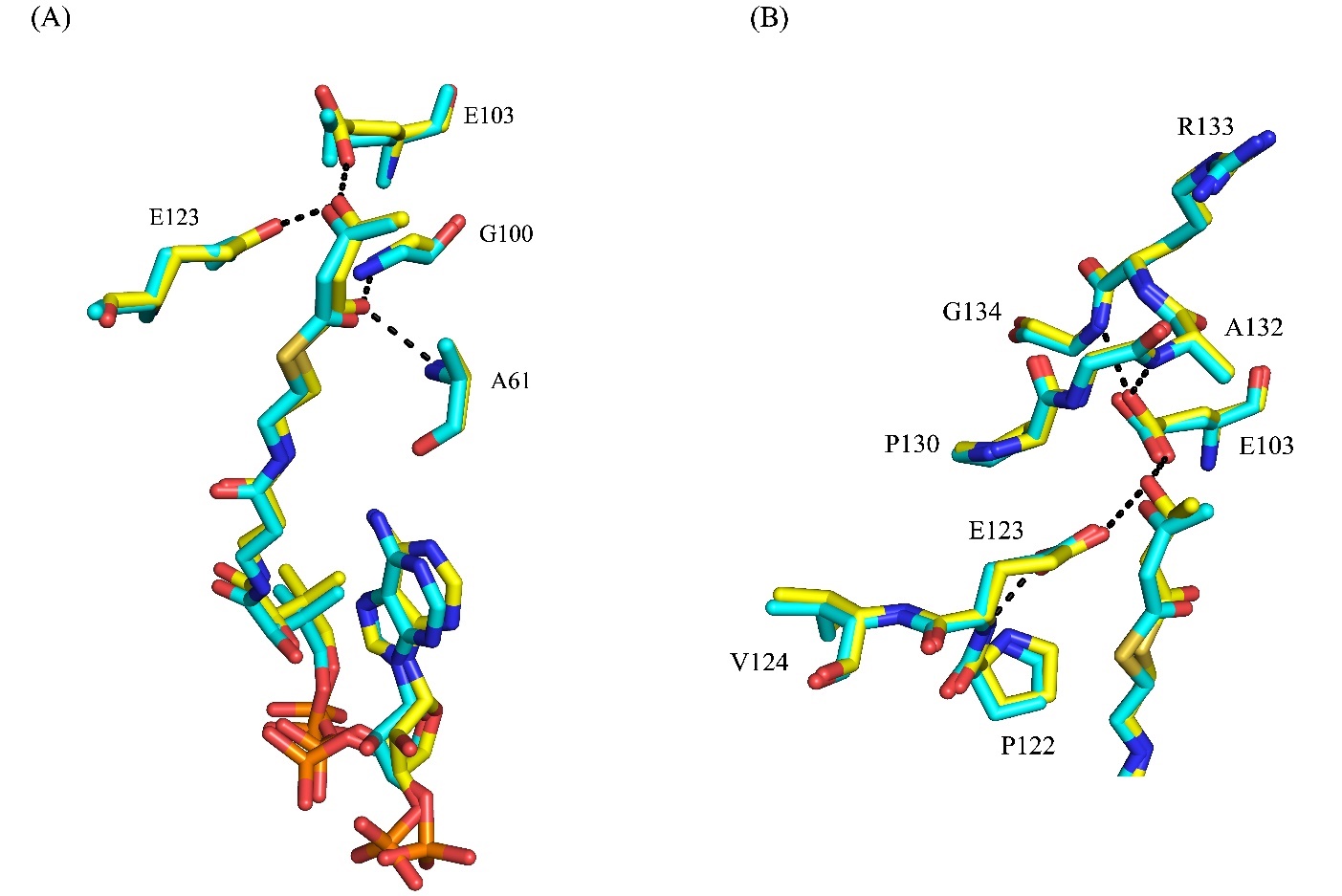


**Fig. S4. The geometry of the hydratase active site of the 3OHC4-NAD^+^ structure (obtained from a crystal soaked with 0.2 mM 3*S*-hydroxybutanoyl-CoA and 2 mM NAD^+^) is the same as observed in the structure obtained by cocrystallization in the presence of 2 mM AcAc-CoA and 2 mM NAD^+^ (PDB ID 5MGB).** The ECH active site of the 3OHC4-NAD^+^ structure (PDB ID 6ZIB, molecule A, complexed with AcAc-CoA) is shown in yellow and the active site of the 5MGB structure (molecule A, complexed also with AcAc-CoA) is shown in cyan. The dotted lines visualize the hydrogen bond interactions of the thioester oxygen atom as bound in the oxyanion hole, with N(Ala61) and N(Gly100). Also shown by dotted lines are the hydrogen bond interactions between the catalytic glutamates and the 3-keto oxygen atom of AcAc-CoA.

**
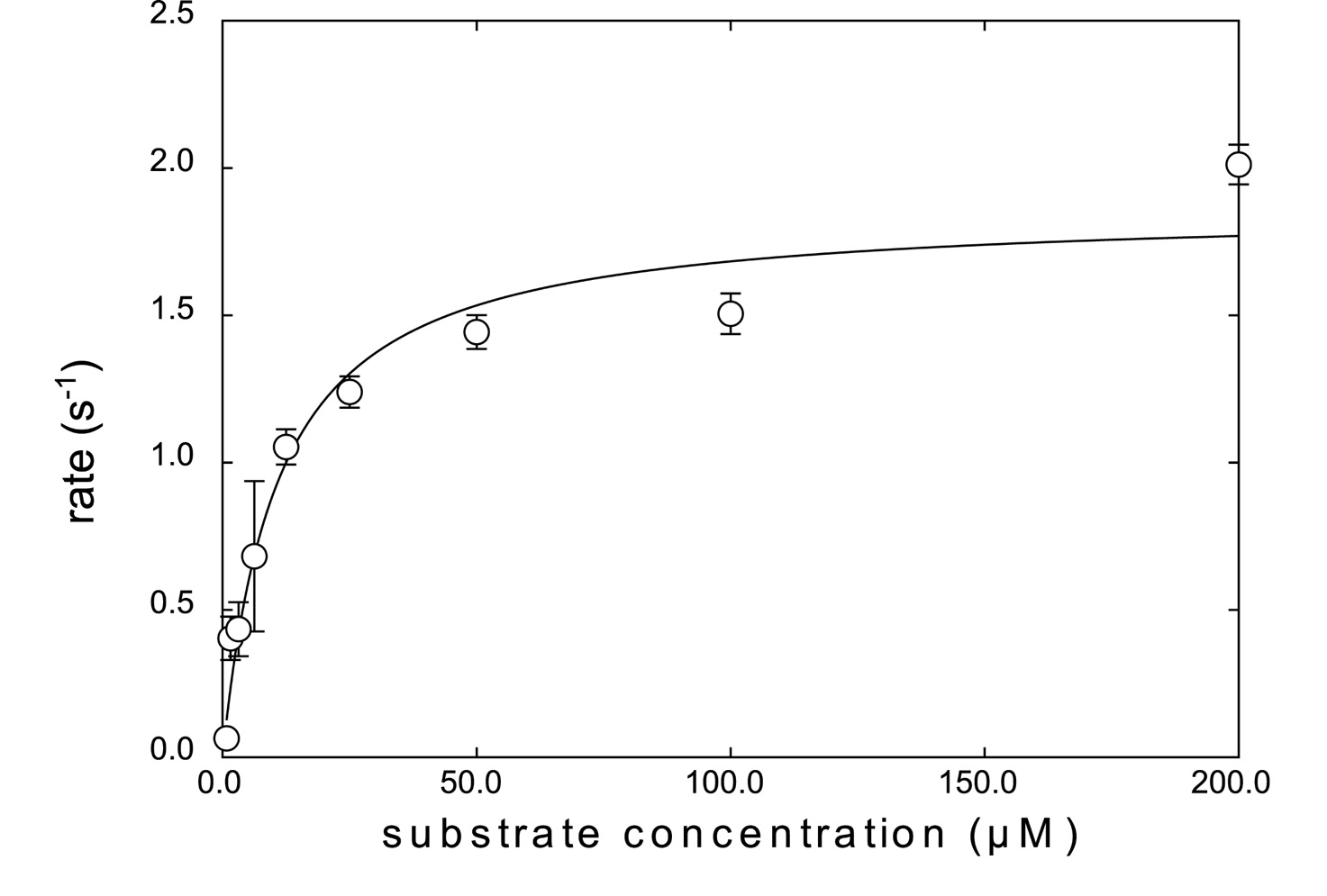
**

**Fig. S5. Steady state kinetics Michaelis-Menten graph concerning the dehydrogenase reaction catalyzed by RnMFE1 for the substrate 3*S*-hydroxybutanoyl-CoA.** In this Michaelis-Menten graph the initial rates are plotted as a function of the 3*S*-hydroxybutanoyl-CoA substrate concentration. All available rate measurements for each concentration are included in this graph. The error bars visualize the range of the measured rates.
